# Low cost, LoRa based river water level data acquisition system

**DOI:** 10.1016/j.ohx.2023.e00414

**Published:** 2023-03-17

**Authors:** Jason N. Kabi, Ciira wa Maina, Edwell T. Mharakurwa, Stephen W. Mathenge

**Affiliations:** a Centre for Data Science and Artificial Intelligence, Dedan Kimathi University of Technology. P.O. BOX, PRIVATE BAG, 10143, Dedan Kimathi, Nyeri, Kenya; b Department of Electrical and Electronic Engineering, Dedan Kimathi University of Technology. P.O. BOX, PRIVATE BAG, 10143, Dedan Kimathi, Nyeri, Kenya

**Keywords:** Water level data, Sensor node power analysis, Sensor node deployment, Wireless sensor networks

## Abstract

In recent years, climate change and catchment degradation have negatively affected stage patterns in rivers which in turn have affected the availability of enough water for various ecosystems. To realize and quantify the effects of climate change and catchment degradation on rivers, water level monitoring is essential. Various effective infrastructures for river water level monitoring that have been developed and deployed in developing countries over the years, are often bulky, complex and expensive to build and maintain. Additionally, most are not equipped with communication hardware components which can enable wireless data transmission. This paper presents a river water level data acquisition system that improves on the effectiveness, size, deployment design and data transmission capabilities of systems being utilized. The main component of the system is a river water level sensor node. The node is based on the MultiTech mDot – an ARM-Mbed programmable, low power RF module – interfaced with an ultrasonic sensor for data acquisition. The data is transmitted via LoRaWAN and stored on servers. The quality of the stored raw data is controlled using various outlier detection and prediction machine learning models. Simplified firmware and easy to connect hardware make the sensor node design easy to develop. The developed sensor nodes were deployed along River Muringato in Nyeri, Kenya for a period of 18 months for continuous data collection. The results obtained showed that the developed system can practically and accurately obtain data that can be useful for analysis of river catchment areas.

## Graphical abstract

**Figure:**
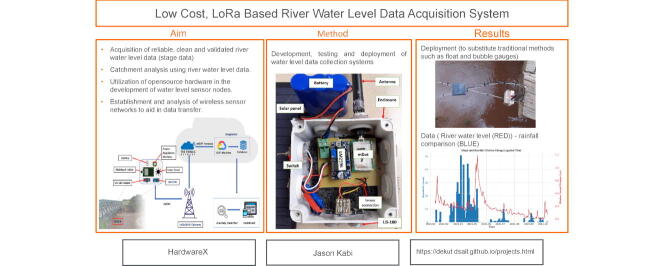

## Specifications Table

**Table:** 

Hardware name	**Project Muringato – River water level monitoring**
Subject area	• Telecommunication Engineering. • Environmental Sciences. • Educational Tools and Open-Source Alternatives to Existing Infrastructure.
Hardware type	• Field measurements and sensors. • Educational Tools and Open-Source Alternatives to Existing Infrastructure. • Telecommunication Engineering
Open-Source Licence	• CC BY 4.0
Cost of Hardware	• Multitech mDot based system = USD 90 (Sensing element included)
Source	https://data.mendeley.com/datasets/mszg723r9b/4
File Repository	https://doi.org/10.17632/mszg723r9b.4

## Hardware in context

Effects of climate change and river catchment degradation have led to an increase in the need for conservation efforts around catchment areas. To boost the effectiveness of these conservation efforts, personnel in organisations tasked with the implementation of various conservation plans and researchers at large, require catchment parameter datasets such as river water level from rivers in various catchment areas to help in research [1], [2]. In addition, skilled personnel involved in development and building of critical infrastructure such as bridges along river channels require river water level data for an extended period to aid in design [3]. Also, water level data or stage data has proved to be a vital tool in hydrology due to its direct relationship with climatic datasets such as rainfall and catchment characteristics like vegetation cover [3].

Stage is defined as the water level in a stream/river with reference to a set datum. The datum is any random point of reference related to the depth of the river channel. Staff gauges, wire gauges, float gauge recorders and bubble gauges are some of the instruments/tools that have been developed by experts over the years to measure and monitor stage [4], [5]. Staff gauges are very common at dams, weirs and stream gauges. A measurement is established by tracing an increase or decrease in stage in contact with a fixed graduated staff. Wire gauges are graduated scale wires which are guided perpendicularly into streams and other water points to make measurements [5]. Float gauge recorders and bubble gauges are more complicated in design and deployment but fairly accurate in measuring stage [5].

Despite having reliable and practically accurate results, traditional instruments such as float gauges, wire gauges, staff gauges and bubble gauges are voluminous and expensive to build, deploy and maintain. These qualities also affect the ability to deploy many of these traditional gauging instruments since gauging of a stream is based on the importance of a stream to the ecosystem. Also, sizable systems cannot be moved around, a fact which makes monitoring at different locations challenging. In addition, these traditional instruments do not possess telemetry capabilities. Lack of data transmission methods leads to human involvement which can in turn introduce errors in the data collected via manual recording. By harnessing the capabilities of technology, research can be advanced in areas of development and deployment of cheap, small and automated stage monitoring devices that can send data remotely via wireless sensor networks not limited to LoRaWAN, SigFox, and GSM, etc [6].

The water level monitoring system sensor node developed and outlined in this paper was based on a fast-prototyping platform namely: the Multitech mDot 868 MHz. Using an ultrasonic sensor connected as a peripheral component and an onboard LoRaWAN transceiver, the platform was programmed to collect and transmit stage data. The Multitech mDot is an Arm® Mbed™ programmable LoRa module from Multitech which is ideal for fast prototyping [7]. The module is programmed using the Mbed platform (Mbed cli – Mbed command line programming tool) which allows development of software in C/C++ and also provides drivers/libraries for the peripheral devices connected to the microcontroller unit [8], [9].

Although microcontroller platforms such as the Multitech mDot have greatly aided researchers in different fields of study to develop monitoring and data collection hardware quickly and at lower costs, the platforms still require a high-level understanding of microcontrollers and coding. Furthermore, to make them useful, they need to be attached to custom hardware that can only be developed by skilled electrical hardware designers

This research describes the development of a stage monitoring system that is cost effective, simple in design and requires knowledge of coding and electrical assembly. The sensor node build relied entirely on off-the-shelf components that can be easily mounted on a fabricated circuit board, reducing the prototype size, assembly time and mastery required to produce a working prototype. The data output of the node developed was reliable and of high quality. The system establishes new minimums in cost, size and assembly of water level monitoring hardware. The development of this system also provides sufficient control capabilities to the research community since monitoring and evaluation of the stage is done remotely using wireless sensor networks.

## Hardware description

The Multitech mDot based sensor node described was built on a 7.3406 cm by 5.4610 cm etched circuit board. The major components on the circuit board were: a DC power supply circuit, a US-100 precision range finder ultrasonic sensor unit, a Multitech mDot and a battery voltage sensor circuit. The system is designed to collect water level data or stage along a river channel using the ultrasonic element. The US-100 has an upper limit of 450 cm hence the sensor cannot be used for river channels which are deeper than 450 cm.

The developed sensor node was powered using a 3.7 V-6600mAH lithium-ion battery which was connected to the custom circuit board using durable sockets. The battery was also interfaced with a solar charging system. The 18 V-5 W solar panel was first connected to a voltage regulation module (LM2596) which was being used to buck the voltage from 18 V to 4.9 V. The LM2596 maintains a constant voltage and current output given a maximum input from the solar panel. On the circuit board, the output pin of the LM2596 was then interfaced with the battery terminal via a 1A, 100 V silicon diode which was reducing the voltage level from 4.9 V to 4.2 V, which is ideal for charging the battery. Furthermore, a diode prevents back feeding. Taking into consideration the intermittent nature of solar energy, the solar charging system was designed to act as a top-up system for the connected battery. A solar panel with a higher voltage and current output could have been used but the battery capacity, system power consumption and the overall system design had to be considered. After testing and deployment, it was concluded that the output current from the 18 V–5 W solar panel was practically sufficient to keep the battery topped-up, since the designed system was not drawing a lot of power from the source. Battery voltage data collected by the on-board voltage sensor proved crucial in keeping track of the battery voltage level and in monitoring the recharging process.

To facilitate data transmission, the mDot is fitted with an on-board LoRaWAN transceiver (915 MHz or 868 MHz). The on-board transceiver attachment contributed greatly to the size-reduction of the custom circuit board which proved to be advantageous during deployment. Besides LoRaWAN, other IoT/sensor communication infrastructure such as GSM or SigFox can be used in data transmission. To connect to these networks, other microcontroller platforms such as the Arduino Nano fitted with adapters tailored specifically for these networks can be considered. The Multitech mDot is specifically built for the LoRaWAN network.

Before test and deployment, the firmware was uploaded onto the mDot using the Mbed CLI. The sensor nodes were also registered on The Things Stack (Network), a network server, where data sent through the LoRaWAN network is received. The Things Stack (Network) (TTN) enables developers to register devices, collect data and reroute the data to other destinations. In this study, the timeseries water level data and the time series battery voltage data collected was being rerouted to a time series database in a Google Cloud machine.

After development, the sensor node circuit boards were fitted into 100 mm*100 mm*70 mm IP65 Acrylonitrile Butadiene Styrene (ABS) plastic, waterproof enclosures to complete the design and facilitate deployment. The components left on the outside were the sensing element, the toggle switch, the antenna and the solar panel to facilitate data collection, system reboot, communication and charging, respectively. The plastic enclosures were then attached firmly to metallic support structures and deployed by securing the metallic structures to bridges and other rigid structures along the target river. The shape of the metallic support structure was dependent on the deployment design and the type of rigid structure available.

Due to factors such as vandalism, device disturbance, power limitations and changes in weather patterns sensor data might contain anomalies that can be discarded using various statistical and classical machine learning algorithms. In this work, the anomalies present in the data collected were detected and eliminated using fine-tuned classical machine learning algorithms such as kernel density estimation (KDE), support vector machine (SVM) and k-means clustering to mention a few [10], [11], [12]. Currently, data processing tasks such as anomaly detection in IoT data are performed after the data have been stored in cloud or local databases. The development of systems such as the one outlined in this paper, can lead to the development of more sophisticated systems capable of local data processing – Edge Processing – where the data is processed at the sensor node level [13]. Monitoring the power being utilised by IoT devices may also help designers to estimate the power requirements of edge processing devices, the viability of IoT devices in the field and whether IoT devices can be suitable for long term monitoring practices if protected and maintained.

The deployed sensor node system described was programmed to collect and transmit river water-level samples after every 5 min. This setup produced a large, high resolution time series, for example, in a 24-hour period, the system was expected to collect 288 samples.

## Design Files

Outlined in Table 1 are design files for the sensor node described in this paper. The file-highlight includes firmware files to run the water level data collection process and Kicad project folders that hold circuit board design files for the sensor node.

I. ***mdot.pro***: Kicad project file that links a PCB file, a schematic file and design library files for the main circuit board design (“Antenna connection” and “Overall (Main) circuit board”). (Kicad schematic and PCB libraries are included in the respective folder).
II. ***extension_soc.pro***: Kicad project file linking a PCB file, a schematic file and design library files for the US-100 ultrasonic sensor extension socket which aids in creating an extended connection between the US-100 and the main circuit board (mdot.pro) (“Component placement” Section). (Kicad schematic and PCB libraries are included in the respective folder).
III. ***depth.cpp***: A Multitech mDot program (C/C++ code) that dictates the data collection and transmission procedure by linking the connected peripheral (US-100) to the controlling unit (mDot) and executing the data collection process (“LoRaWAN network establishment” Section) (Peripheral and control unit libraries included are in the respective folder).
IV. ***formatter.js***: TTN (The Things Stack) payload formatter JavaScript code used in decoding the payload bytes sent by the sensor node through the LoRaWAN network into coherent data. The code is hosted on the TTN application terminal that holds the registered sensor node and the user has to activate it manually for the decoding to happen. (“Energy consumption modelling” section).

**Table 1:** Design file summary.

Design file	File type	Open-source licence	Location of the file
mdot.pro	Kicad project file (.pro file). Schematic & PCB included.	CC BY 4.0	https://data.mendeley.com/datasets/mszg723r9b/4
extension_soc.pro	Kicad project file (.pro file). Schematic & PCB included.	CC BY 4.0	https://data.mendeley.com/datasets/mszg723r9b/4
depth.cpp	Mbed C/C++ file	CC BY 4.0	• mdot-waterlevel-software.
			https://data.mendeley.com/datasets/mszg723r9b/4
formatter.js	TTN Payload formatter	CC BY 4.0	https://data.mendeley.com/datasets/mszg723r9b/4

## Bill of materials

Outlined in Table 2 are the materials required to develop the sensor node. Each component has a designator that identifies it in the build instruction text. The source of materials column highlights the source of materials in the local Kenyan market whereas the material type column highlights the type of material being utilized. Components without the M−designator are used in the realization of the main circuit board build whereas the components with the M−designator are peripheral attachments and accessories used in the realization of the complete deployment-ready design. The components in the first four rows derive their designators from the PCB design file (Table 1: mdot.pro) where their footprints are identified using the designators provided, as shown in Fig. 5.

**Table 2:** Bill of Materials. Components Indicted with (*) are Sold in Bulk which Provide Excess Pieces, Prices are in Accordance with The Local Kenyan Market.

Designator	Component	Number	Cost in USD	Source of materials	Material type
D1	1 N4002 1A 100 V Rectifier Diode*	1	$0.04	Digi-key electronics	Electronic
J1, J3	2PIN Right Angle Plug-in Terminal	4	$1	Pixel Electric	Non-specific
U2	Multitech mDot™ MTDOT-868-X1-SMA-1	1	$50	Digi-key electronics	Electronic
U1	LM2596 DC-DC Buck Converter Step-down Power Module	1	$1.66	Nerokas	Electronic
A1	Header Pin Male Straight − 2.0 mm Pitch	1	$0.17	Nerokas	Non-specific
A2	Header Pin Female Straight − 2.0 mm Pitch	2	$0.34	Pixel Electric	Non-specific
A3	24 AWG Insulated Single Core Copper − 1 Meter	1	$0.5	Nerokas	Non-specific
A4	24 AWG Insulated Stranded Wire − 1 Meter (BLACK)	1	$0.9	Nerokas	Electrical
A5	24 AWG Insulated Stranded Wire − 1 Meter (BLUE)	1	$0.9	Nerokas	Electrical
A6	24 AWG Insulated Stranded Wire − 1 Meter (YELLOW)	1	$0.9	Nerokas	Electrical
A7	24 AWG Insulated Stranded Wire − 1 Meter (RED)	1	$0.9	Nerokas	Electrical
M1	Copper Clad Board Single Sided 10x15 − 1.6 mm	2	$2	Pixel Electric	Non-specific
M2	Lithium-Ion Battery Pack − 3.7 V 6600mAh	1	$11.67	Nerokas	Non-specific
M3	Solar Cell (Panel) 18 V 5 W	1	$6.64	Pixel Electric	Non-specific
M4	US-100 High Precision Ultrasonic Range Finder	1	$2.08	Nerokas	Electronic
M5	LoRa Antenna 868Mhz RF SMA Male	1	$5	Nerokas	Non-specific
M6	(ON)-OFF-(ON) momentary toggle switch	1	$0.6	Pixel Electric	Non-specific
M7	IP65 Waterproof Enclosure 100x100x70mm	1	$3.32	Pixel Electric	Non-specific
M8	Grey RTV Silicone Sealant (weather proofing)	1	$4.55	Jumia Kenya	Non-specific
M9	(3/8″ x 2 1/2″) Full Thread 3/8 Inch Mild Steel Hex Bolt (with nut and washer)	1	$0.3	–	Mechanical
M10	1′(ft), painted, prefabricated mild steel flat bar (1″ * 1/8′’). with drill holes at 3-inch interval to accommodate the coupling bolt (M9) and mounting nails	1	$4	–	Mechanical

The shape and make of the metallic support piece needed during deployment depend on the deployment design and deployment location but in this paper the vertical deployment design of the sensor node is considered as shown in Fig. 12.

## Build Instructions

This section outlines the steps used to construct and assemble the mechanical and electrical/electronic parts of the sensor node. “Electrical/electronic hardware construction and assembly” Section covers the development of the electrical hardware and also gives a detailed description of the sensor hardware and the circuit board design. Section 5.2 discusses the inclusion and attachment of the components that aided in the deployment of the sensor node.

**Fig. 1:**
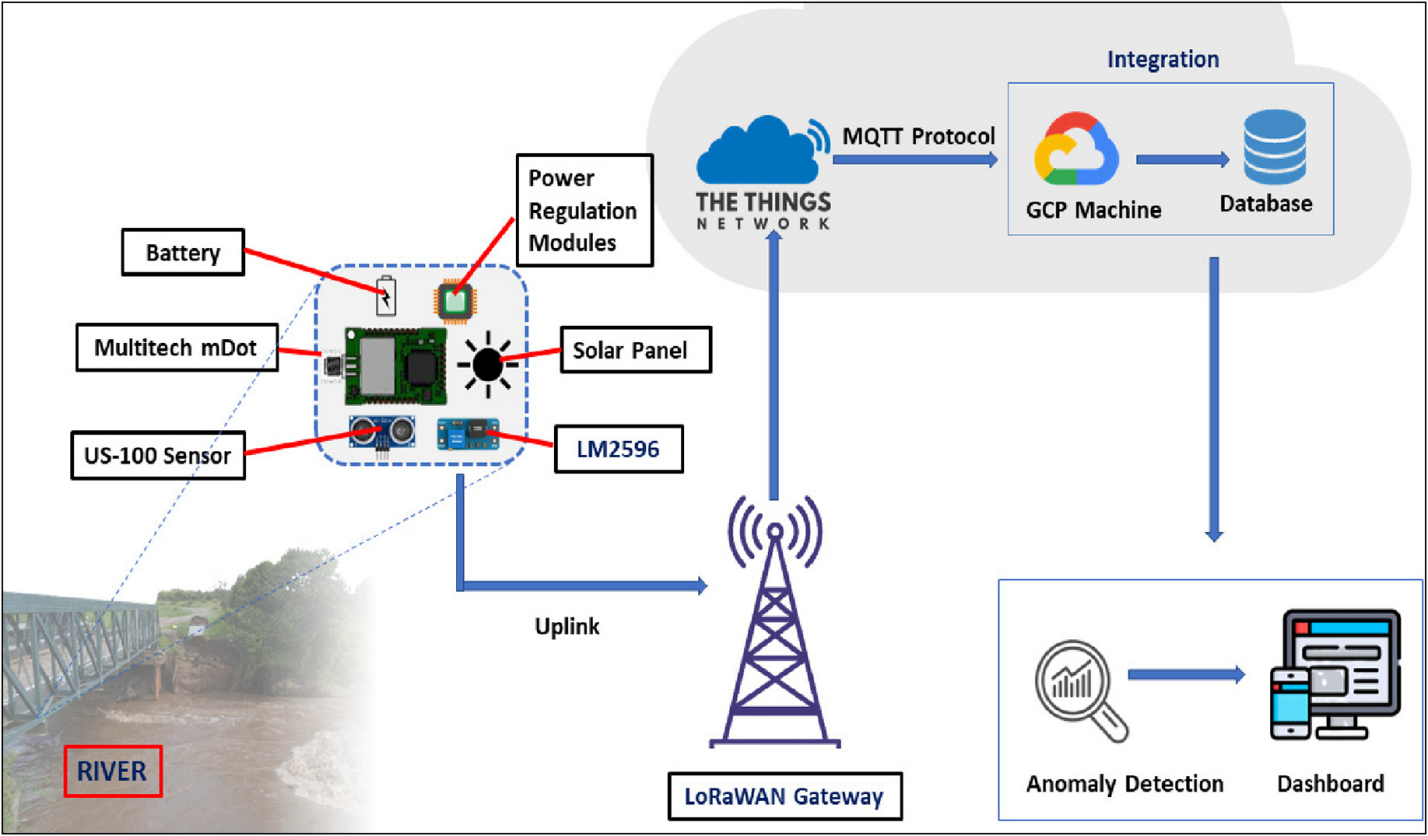
Path Followed by The Data Collected by The Multitech Mdot Based Sensor Node.

Fig. 1 shows the sensor-database path followed by the data collected by the sensor node developed. The data was being transmitted via the LoRaWAN network from the sensor node to the network server (The Things Stack) where it was being decoded using the formatter.js code (Table 1) provided and then rerouted to a permanent database on a Google cloud Engine. The rerouting was being done by executing an MQTT protocol - INFLUX data transfer code on the Google Cloud Engine. The MQTT protocol was being brokered by the Things Stack (TTN) which enabled the engine to communicate with it and scrap the available decoded data the moment it was sent to the network’s server device terminal.

### Electrical/electronic hardware construction and assembly

This section outlines the electrical hardware utilized in developing the electrical/electronic build of the sensor node. The outline is divided into subsections which highlight the builds of the various components used and the schematics which show the required connections between components. The final build is established by cascading components highlighted in the subsections of “Electrical/electronic hardware construction and assembly” Section. The schematic form used in highlighting the connections is clear and can be used by developers in the realization of the device on a prototyping board (breadboard). The schematics are from Kicad [14], a free software suit used in the design of circuit boards, which makes them transferrable. The Kicad design files details are provided under Section 3: Design files.

#### Ultrasonic sensor (US-100)

**Fig. 2:**
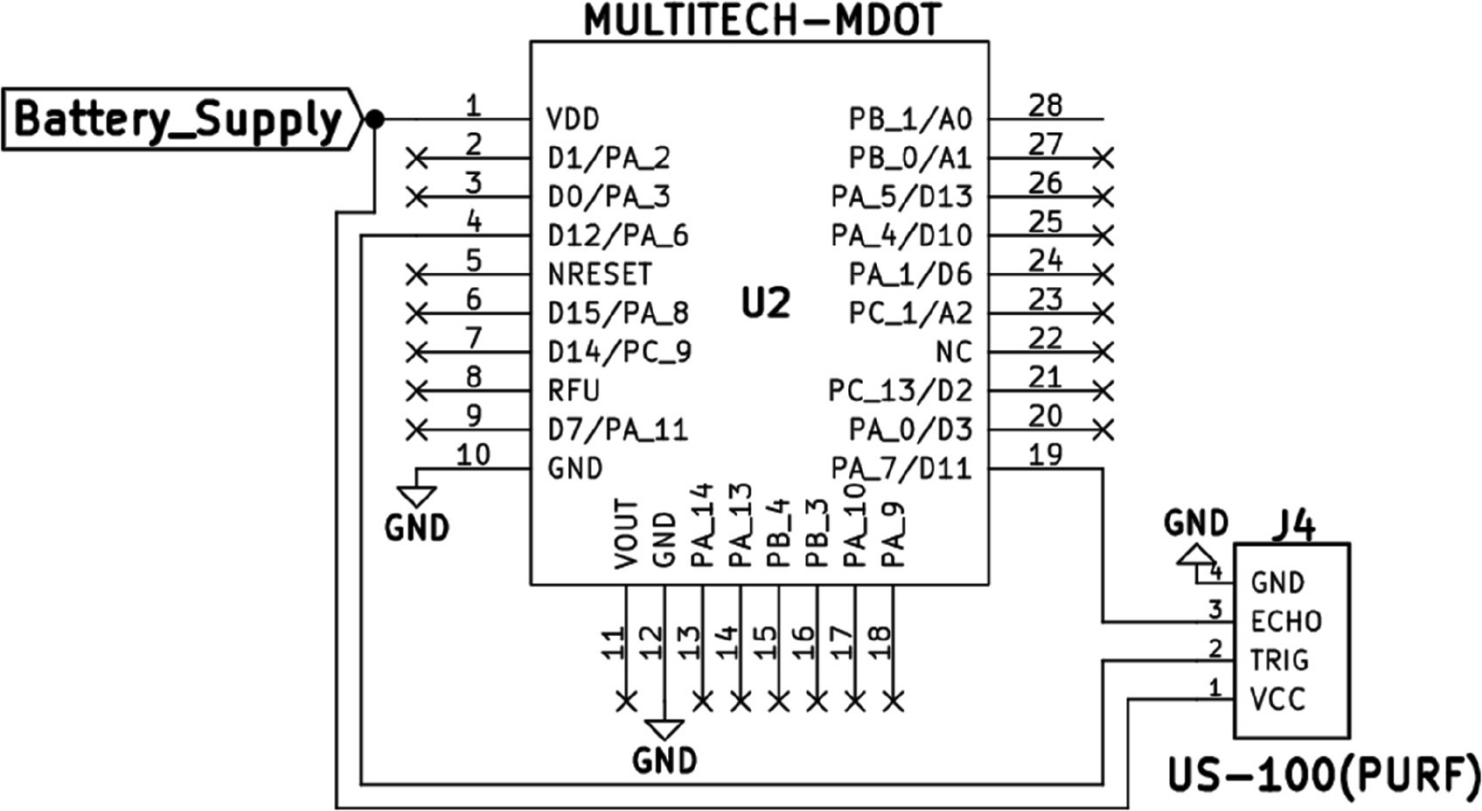
Connection Between the Multitech Mdot (U2) and the US-100 (PURF)(M4) on the Custom Circuit Board Build. (Kicad Schematic).

An ultrasonic sensor measures distance to an object using ultrasonic sound waves. The ultrasonic sensor used in this work was the US-100 (M4-J4) which is stated to provide a 2 cm-450 cm non-contact measurement function with an accuracy of about 3 mm. To start ranging, the sensor is supplied with a short 10us pulse to its trigger terminal by a programmable microcontroller. The sensor is then able to send out an 8-cycle burst of ultrasound at 40 kHz to measure the distance. To complete the ranging, the sensor receives the echo caused by the target obstacle (in this case the water surface) on its echo terminal. The distance to the obstacle is then calculated using the time interval between the sending trigger signal and the receiving echo signal. Fig. 2 shows the connection between the US-100 and the Multitech mDot (U2) which was the main controlling unit utilized in this work.

Any power source with a voltage output between 3 V and 5 V (Lithium-ion Battery (M2)) can be used to run the US-100, hence no logic converters and dividers are needed in the circuitry. There are two modes of operating the US-100; the “HC-SR04 mode” and the “serial-UART mode”. For the sensor to be operated in the HC-SR04 mode, the jumper present on the sensor PCB has to be disengaged. In this work, the US-100 was utilized in the HC-SR04 mode (trigger and echo pin enabled). The US-100 has a 5-pin (standard 2.54 mm) interface. The VCC and the 2 GND terminals are used in powering the sensor, whereas the ECHO and TRIGGER pins are used in data collection. On Fig. 2, the 2 GND pins are shown as one since they are interconnected on the sensor PCB.

In the firmware provided (Section 3: Design files: depth.cpp - HCSR04.h) and as shown on Fig. 2, the Trig pin on the US-100 was assigned to pin D12 on the mDot. The Echo pin was assigned to pin D11. The pin assignment followed the layout utilized in the open-source library – US100-HCSR04 – used in distance calculation. The US100-HCSR04 library held the timer function code that was utilized in calculating the distance based on the inputs from D11 and D12.

Using the timer function, the trigger pin (D12) is first cleared and set to - HIGH to produce the needed 10us pulse. The reading of the echo pin begins the instant the pulse is sent out. The timer function reads the travel time of the echo pulse which is the time interval between the sending trigger signal and receiving echo signal and the result is then stored as variable = **duration**. The duration variable is given in microseconds and is utilized in the calculation of the proximity of the target obstacle. To enable the process, the US-100 had to be powered, hence the VCC pin was connected to the positive terminal of the DC power source (M2) being utilized and the GND terminals were connected to the negative terminal.

#### Power supply system layout

Table 3 highlights the power sources for the electronic components used in the realization of the sensor node build. The table also outlines the solar capacity installed to top-up the battery charge.

**Table 3:** Power Source for The Electronic Components and The Solar Capacity in Use.

Component	Power Source
Multitech mDot	Lithium-ion battery- 6600mAh (M2)
US-100	Lithium-ion battery- 6600mAh (M2)
Solar capacity	18 V/ − 5 W (M3)
Charging voltage	4.2 V

**Fig. 3:**
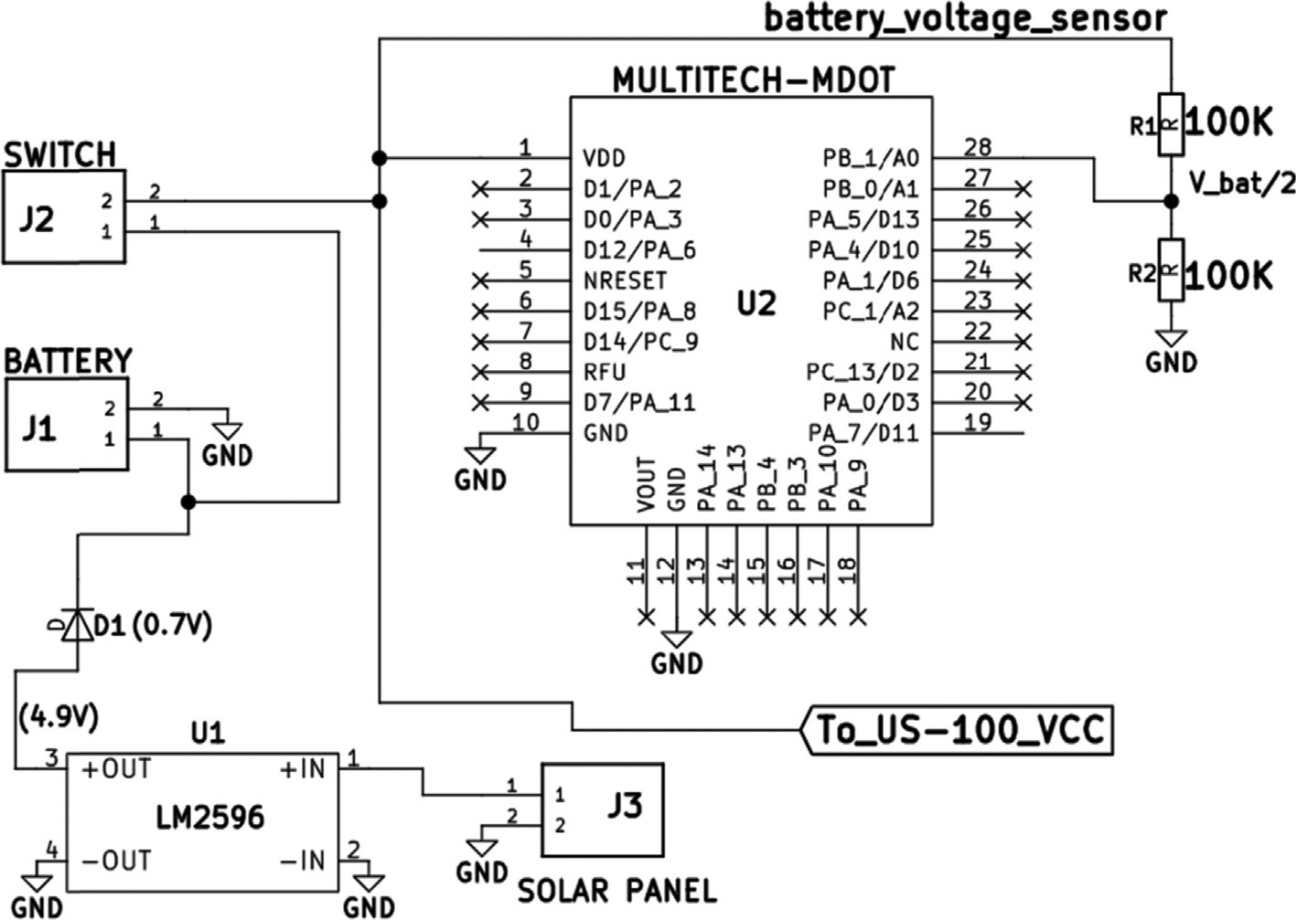
Battery Voltage Sensor Layout and The Power Supply System Layout.

As shown in Fig. 3, the Multitech mDot was powered through Pin 1 – VDD. Like many microcontrollers, the mDot has more than one GND pins (Pin 11 and Pin 12) which were connected to the negative terminal of the power source. The required positive DC voltage supply at VDD-mDot was stated as a range between 3.0 V and 5.0 V hence the lithium-Ion battery was fit to supply the needed power. Since the amount of energy available in Lithium-Ion packs is finite, the batteries needed to be charged regularly. Retrieving batteries from deployed sensor nodes was tedious and disruptive, hence an alternative method of charging – solar energy – was considered in this study.

A solar panel was used to charge the 6600mAH battery. As shown on Fig. 3, the solar panel was connected to the battery terminals via an LM2596-diode combination to aid in solar charging-voltage regulation and prevention of back-feeding respectively. Charging-voltage regulation was vital since a regulation failure can lead to battery damage.

The US-100 ultrasonic element was also drawing power from the battery as shown on Fig. 2, Fig. 3. The mDot has a V-out Pin (Pin 11: Fig. 2, Fig. 3), which possesses a 3.08 V, 25 mA output that can be used in powering the sensor, but drawing power from this pin can be disadvantageous since the output depends on the amount of power the mDot is drawing and the amount available in the power source. Therefore, connecting the sensor directly to the power source was a better layout.

In most IoT practices, where data transmission through networks is required, transmission test runs before deployment are important. In most cases, a transmission test is achieved by a battery disconnection-connection procedure which triggers the control unit to collect and send data through the network. The disconnection-connection is tedious and can cause stress on the battery connectors (J1 or J2) used in connecting the battery to the main circuit board. To avoid stressing or damaging the connectors, a toggle switch (M6) which connects and disconnects the connected power source is crucial. By toggling the lever, the disconnect-connect procedure is achieved without disassembling the sensor node. In this work, the switch present between the hardware and the battery (Fig. 3) was meant to enhance the disconnect-connect procedure and also facilitate manual reboot and shutdown during testing and deployment.

#### Battery voltage sensor

In IoT, electrical power is limited hence a lot of energy conservation practices such as sleep mode execution and peripheral device power down are aimed at conserving it. To keep track of the amount of power available for the sensor node, various on-board power analysis sensors can be developed and deployed. Shown in Fig. 3 is a battery voltage sensor that was connected to the battery terminals. The sensor was composed of a voltage divider and a connection to one of the analog pins on the mDot (A0). During water level data collection, the microcontroller was also sampling the battery voltage level using the battery voltage sensor layout and sending the voltage value through the network. The Multitech mDot analog pins are 5 V- tolerant but to avoid feeding high voltage signals to pin A0, the signal from the battery was first connected to a voltage divider (R1 & R2) and then interfaced with pin A0.

#### Antenna connection

Since the mDot has an onboard LoRaWAN transceiver, the antenna socket was also onboard. The recommended antenna for the mDot socket was a 3dBi 868 MHz off-the-shelf LoRa antenna (19.5 cm length, 1.3 cm diameter) connected as shown in Fig. 11.

#### Overall (Main) circuit board

**Fig. 4:**
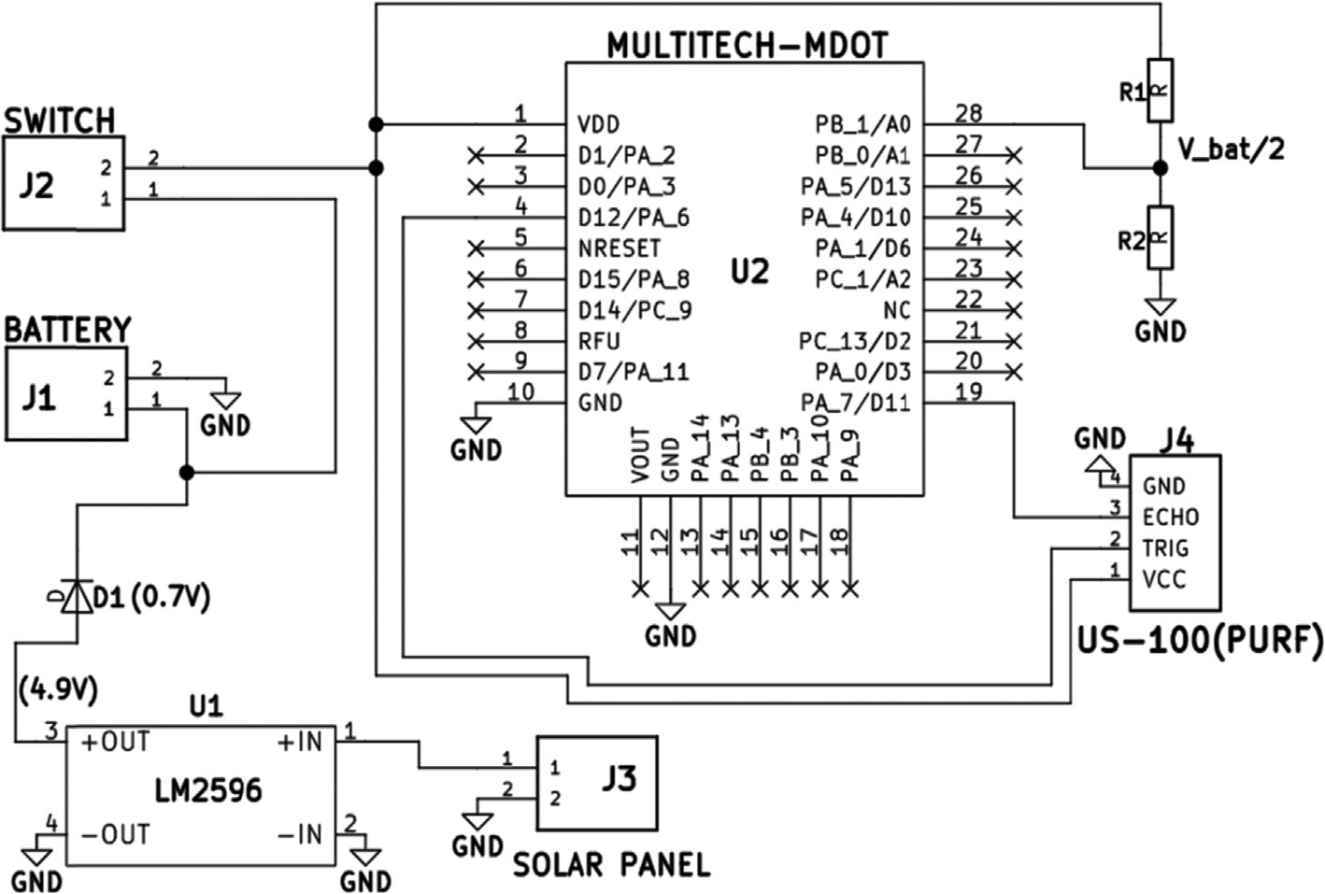
Overall Schematic: A Combination of The Component Schematics (Sensor Connection (5.1.1), Power Supply System (5.1.2) Battery Voltage Sensor (5.1.3)).

**Fig. 5:**
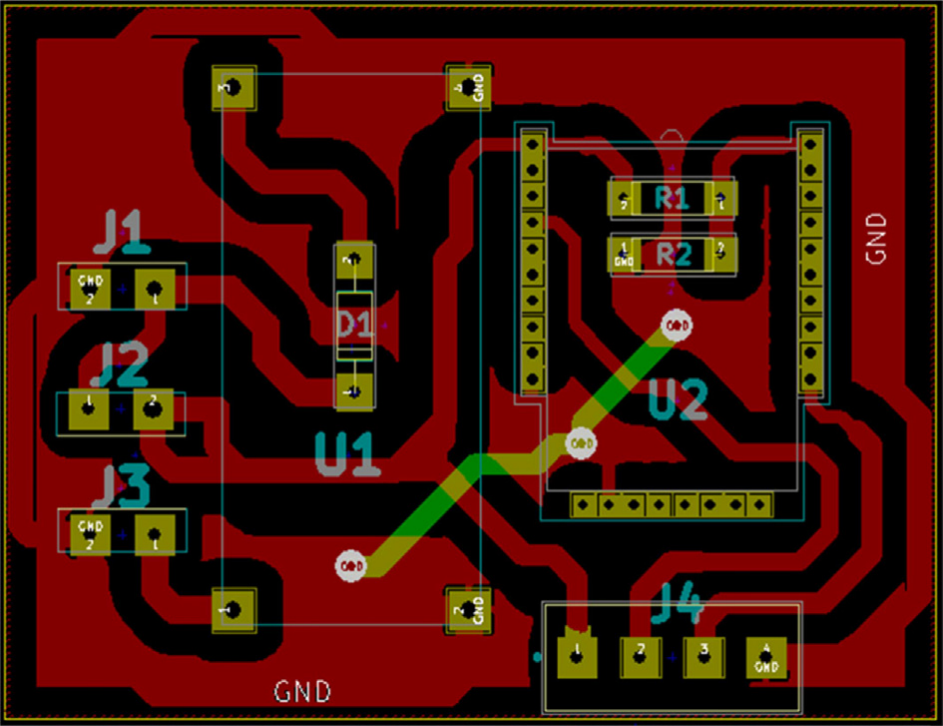
Sensor Node Main Circuit Board Design.

**Fig. 6:**
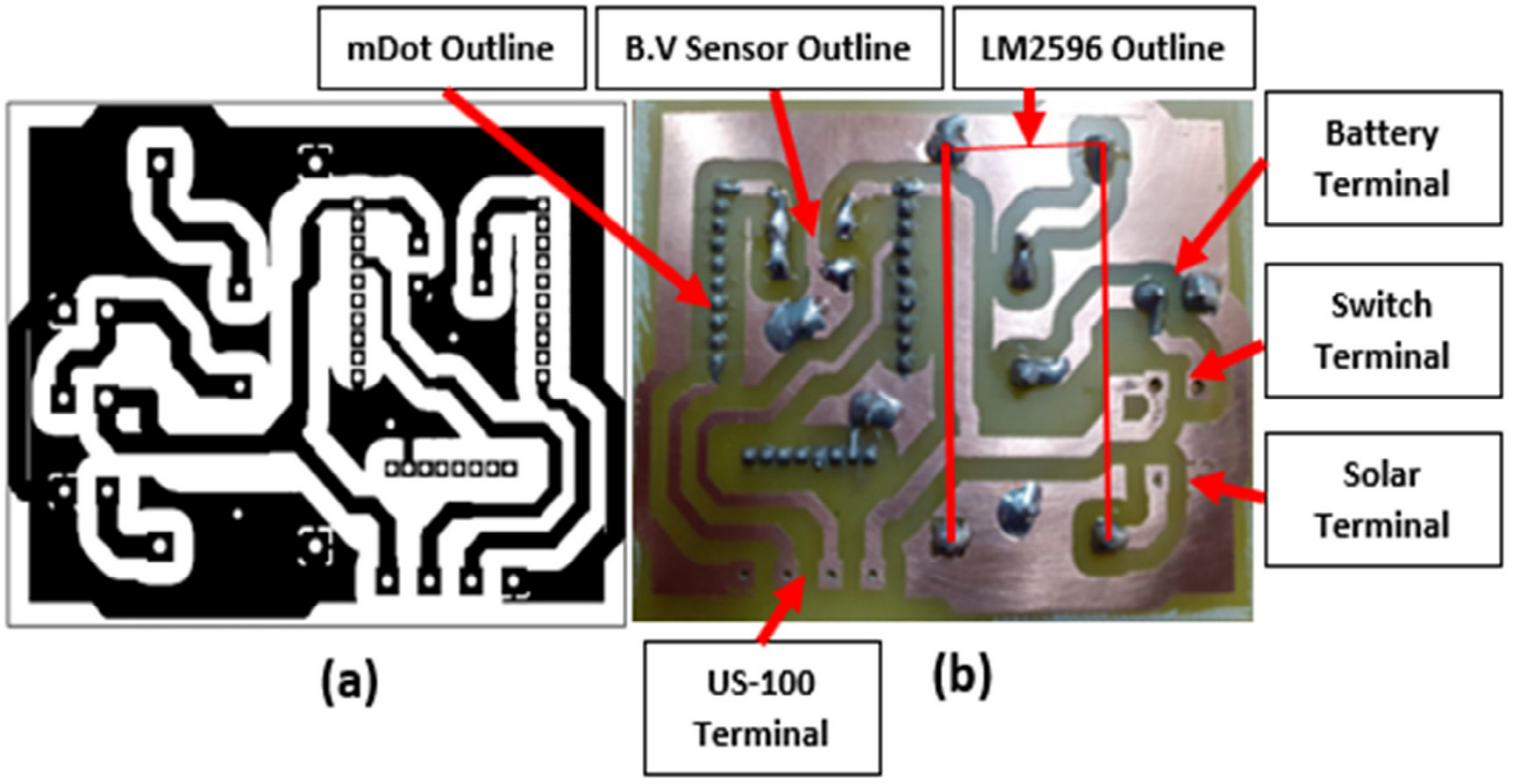
(a) PDF Printout (Track Mask) on a Glossy Paper and (b) The Final Product After Etching (B.V Sensor = Battery Voltage Sensor).

Fig. 4 shows the complete Kicad schematic diagram used to develop the overall circuit board design shown in Fig. 5, Fig. 6-a. The schematic is a combination of the component schematics (Sensor Connection (**5.1.1**), Power Supply System (**5.1.2**) Battery Voltage Sensor (**5.1.3**)). The Kicad PCB design is depicted in Fig. 5 and the final etched product is shown on Fig. 6-b. The design files mentioned and shown in Fig. 4, Fig. 5 can be accessed by executing the provided mdot.pro folder on Kicad 4.0.7.

Fig. 5 outlines the circuit board component footprints that were used in connecting the main components to the circuit, which is in turn represented by the red and green tracks. The GND terminal connected to all GND pins, was designed as a copper zone and the other connections were made using copper tracks. The power tracks (2 mm) were thicker than the signal tracks (1.5 mm). The green track which cuts through some of the red tracks represents a GND jumper which was used to connect the unconnected GND zones, during component placement the jumper connection was made using a 24 AWG Insulated Single Core Copper wire (A3). J2 and J4 are not included in the Bill of materials (Table 2) since the switch and sensor connection was not made using the terminal connectors as shown in Fig. 11. Table 4 shows the matching of the footprint designators to the components.

**Table 4:** PCB design component specification (*: Not represented on the Bill of Materials).

Label	Component
D1	Diode
J1	Battery terminal
J2*	Switch terminal*
J3	Solar panel terminal
J4*	US-100 terminal*
R1	Resistor
R2	Resistor
U1	LM2596
U2	Multitech mDot

#### Overall circuit board realization (ETCHING)

Etching is a subtractive chemical process used in the creation of etched circuit boards. An acid is used to extract undesired copper sections from a ready-made copper laminate (copper clad board (M1)). The etching-ready laminate is created by applying a non-permanent mask that protects parts of the copper face (designed circuit) from the acid and exposes the unwanted copper layer (track gaps). The etching process usually starts with the preparation of a track mask that is identical to the circuit designed on software. Fig. 6-a shows a pdf print out of the track mask identical to the design shown in Fig. 5. The mask forms the circuit as the acid removes the unwanted copper. To create the sensor node main circuit board shown on Fig. 6-b, the steps listed below were followed. The tools and materials necessary are highlighted in the text.

I. The mask was first printed out on glossy paper using a laser printer.
II. The mask dimensions were measured to confirm whether the printing scale was a 1:1 scale (100% of the actual design on software). Normally, if a 1:1 scale is not utilized, the dimensions of the mask do not match the dimensions of the actual design on software hence, the component footprints/sockets tend to shrink, a property that affects the component placement process (“Overall Circuit Board Realization (ETCHING)” Section).
III. Once the mask on the glossy A4 paper passed the checks, it was cut out from the rest of the paper using a pair of scissors and then used to measure the exact size of the copper laminate needed (M1). The piece of copper clad needed was cut out from the rest of the copper board using a desk paper cutter (*Note: match the size of the copper board and the mask*).
IV. The needed copper clad piece was then polished using a piece of steel wool to remove impurities and the oxide layer on its surface. The track mask was then transferred to the polished copper clad (laminate) board by placing it facedown over the board and pressing on it using a hot iron box for toner transfer.
V. After the transfer, the print paper was removed with care to avoid cracks and discontinuities on the transferred track mask, which can translate to discontinuities on the circuit board.
VI. The copper laminate board with the transferred mask was dipped into an aqueous sodium silicate tank in preparation for the etching tank [process – 1 min to 2 min].
VII. After the sodium silicate treatment, the board was rinsed with running water and then dipped into an etching tank filled with Ferric Chloride. (*Protective gear should be worn when dealing with Ferric Chloride*). In the tank, a REDOX reaction dissolved the exposed copper leaving behind the copper tracks covered by the toner mask [process time – 10 min to 20 min].
VIII. After the etching was done, the toner mask was wiped off using acetone and the desired copper tracks were exposed and inspected to make sure there were no discontinuities or short circuit connections present.
IX. The next step after the inspection was drilling. This was done using a hand drill fitted with drill bits of different diameters depending on the size of the drill holes. The drilling also aided in outlining the components’ footprints on the etched circuit board. [Process time 10 min to 20 min]. Fig. 6 shows the mask and the final product after etching.

#### Component placement

**Fig. 7:**
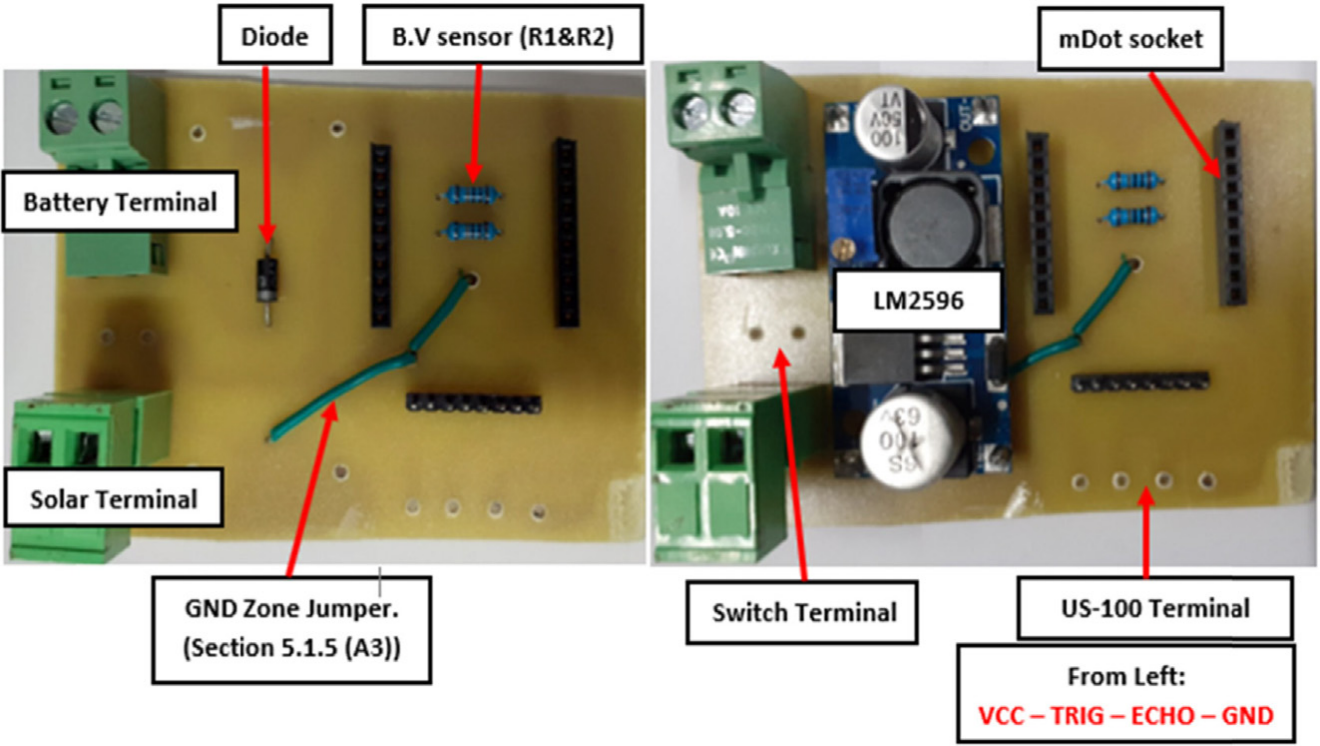
Component placement process. (B.V Sensor = Battery Voltage Sensor).

The last phase of the circuit board realization was component placement. A soldering iron, solder and soldering paste were needed in this phase. Component U2 (Multitech mDot) was purchased with soldered 2 mm male header pins which were matched with corresponding 2 mm female headers pins (A1 & A2) soldered on the etched board for proper operation as shown in Fig. 7. This perfect header pin connection also facilitated an easy mount-dismount procedure during programming.

Normally, when placing/soldering components on an etched board, the smaller components should be considered first since some of them have to be placed under larger components to limit the size of the circuit board. Following the small-first plan, the Diode [D1], Resistor [R1 and R2] and the jumper piece used in connecting the ground planes, were soldered first followed by the U1 (LM2596) which was attached to the board using 1.5 mm (diameter) header pins designed using paper clips. To finish off the assembly the Solar and Battery terminal sockets (J1 & J3) were attached to create the solar and battery connections points. The toggle switch was connected to the board using 24 AWG hook up, stranded wires and fastened to one of the rubber bungs on the node plastic enclosure (“Component Placement” Section), to facilitate quick system reboot and shutdown. Fig. 7 shows the component placement process. The sensor and switch were not connected at this assembly level since they were regarded as peripheral components.

#### Sensor and switch installation

##### Sensor connection

**Fig. 8:**
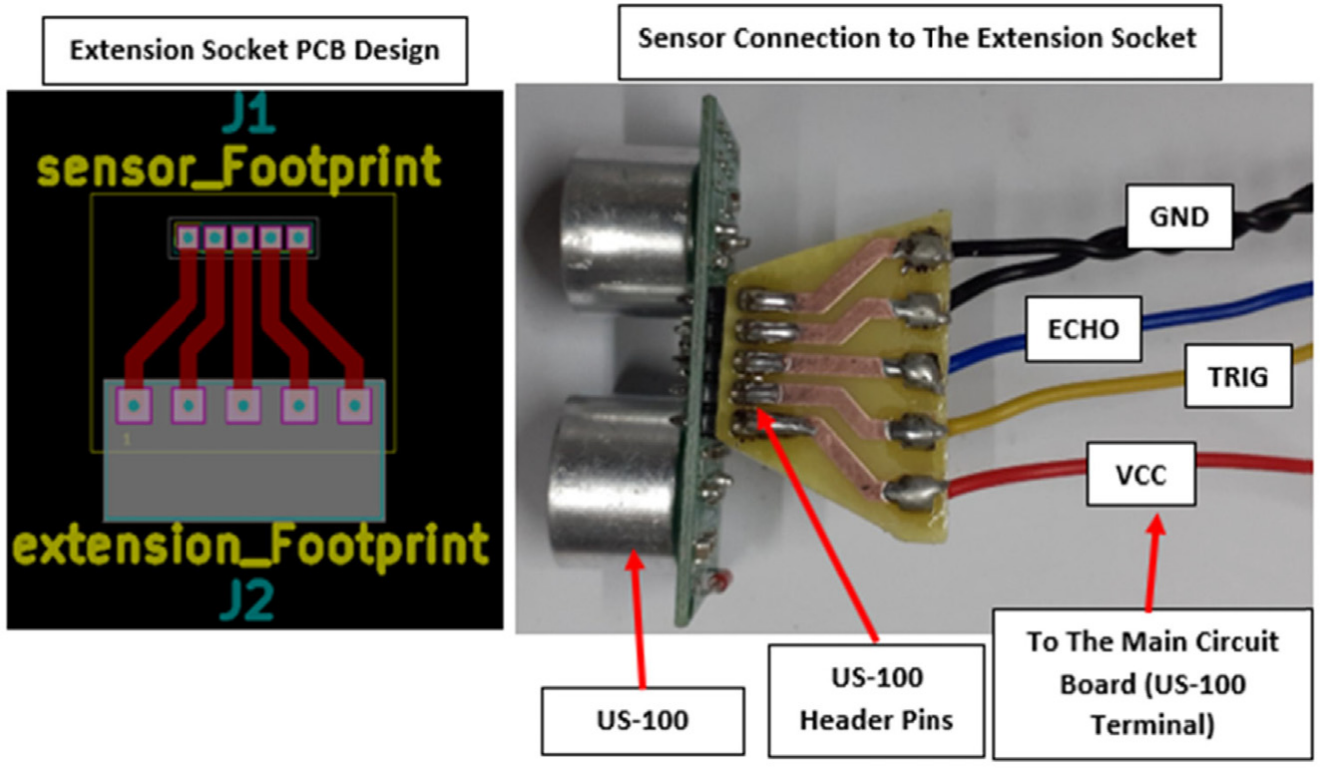
Sensor connection to the extension socket.

To facilitate data collection, the ultrasonic sensor had to be attached to the plastic housing to facilitate the external protrusion of the cylindrical sensing elements. This called for an extended connection between the sensor pins and the US-100 Terminal as shown in Fig. 11. To make this connection, an extension socket with a through-hole footprint that matched J4 (Fig. 8 (US-100 Terminal)) on one side and a footprint that matched the sensor header pins on the other was needed. Stranded hook-up wires interfaced the extension socket with J4 and the sensor header pins were soldered on the opposite side as shown in Fig. 8.

With this set-up, the circuit board and the sensor were separate components connected with wires to facilitate deployment. The connection procedure of the wires followed the pin assignment on the main circuit board and the layout of the sensor header pins. The extension socket was created using the etching process outlined under “Overall (Main) circuit board” Section. The socket design files provided under *Section 3*: **Design Files**.

##### Sensor fitting

**Fig. 9:**
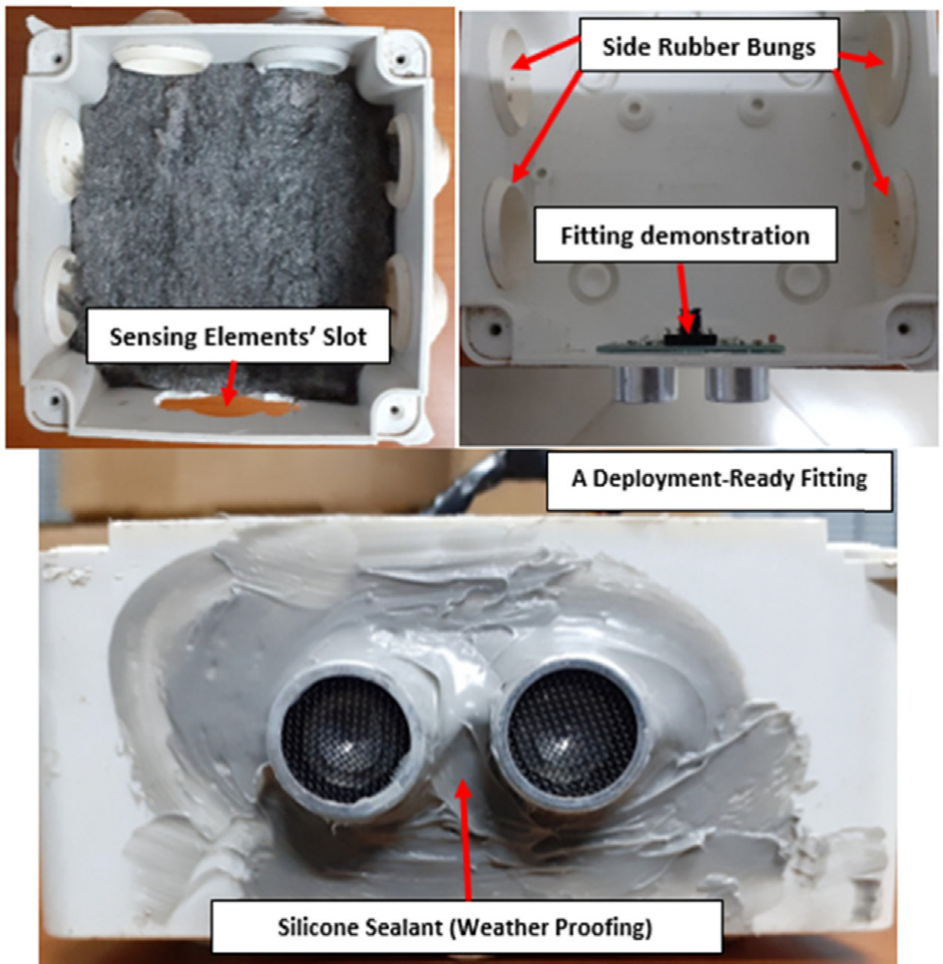
Sensor fitting on the plastic housing.

To enable data collection, the cylindrical elements had to be exposed and face the water surface in the river channel. This was achieved by tracing the outline of the two elements on the width of the IP65 Waterproof plastic Enclosure (100x100x70mm) (M7), cutting out the trace and fitting the elements through the designed opening as shown in Fig. 9. To make the sensor fitting rigid and weather proof, silicon sealant (M8) was used to fill up the spaces between the sensor and the plastic lining. With this setup, the fragile part (custom PCB) of the US-100 was not exposed thus protecting it from harsh weather conditions.

##### Toggle switch

**Fig. 10:**
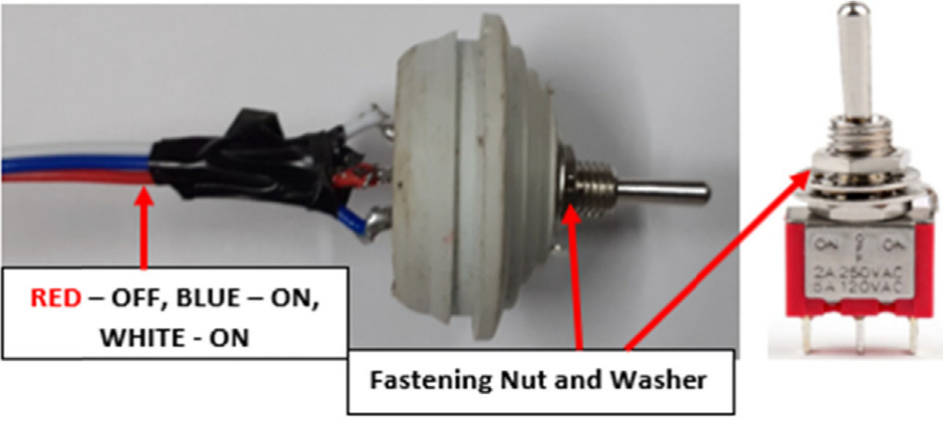
Toggle switch fitting.

The IP65 enclosure used as the main housing unit for the sensor node had 7 removable rubber bungs covering 26 mm diameter holes for external cable entry as shown in Fig. 9. The toggle switch was held in position by one of the rubber bungs and only the toggle lever was external as shown in Fig. 10. To make the fitting rigid, the switch was fastened to the bung using the nut below the lever. Finally, silicone sealant was applied to make the fitting weatherproof. As shown in Fig. 10. The toggle switch used had 3 terminals (ON – OFF - ON). The OFF terminal was connected to the power source (Battery terminals) whereas the 2 ON terminals were connected to the load tracks (mDot and Sensor power tracks) as shown in Fig. 6.

**Fig. 11:**
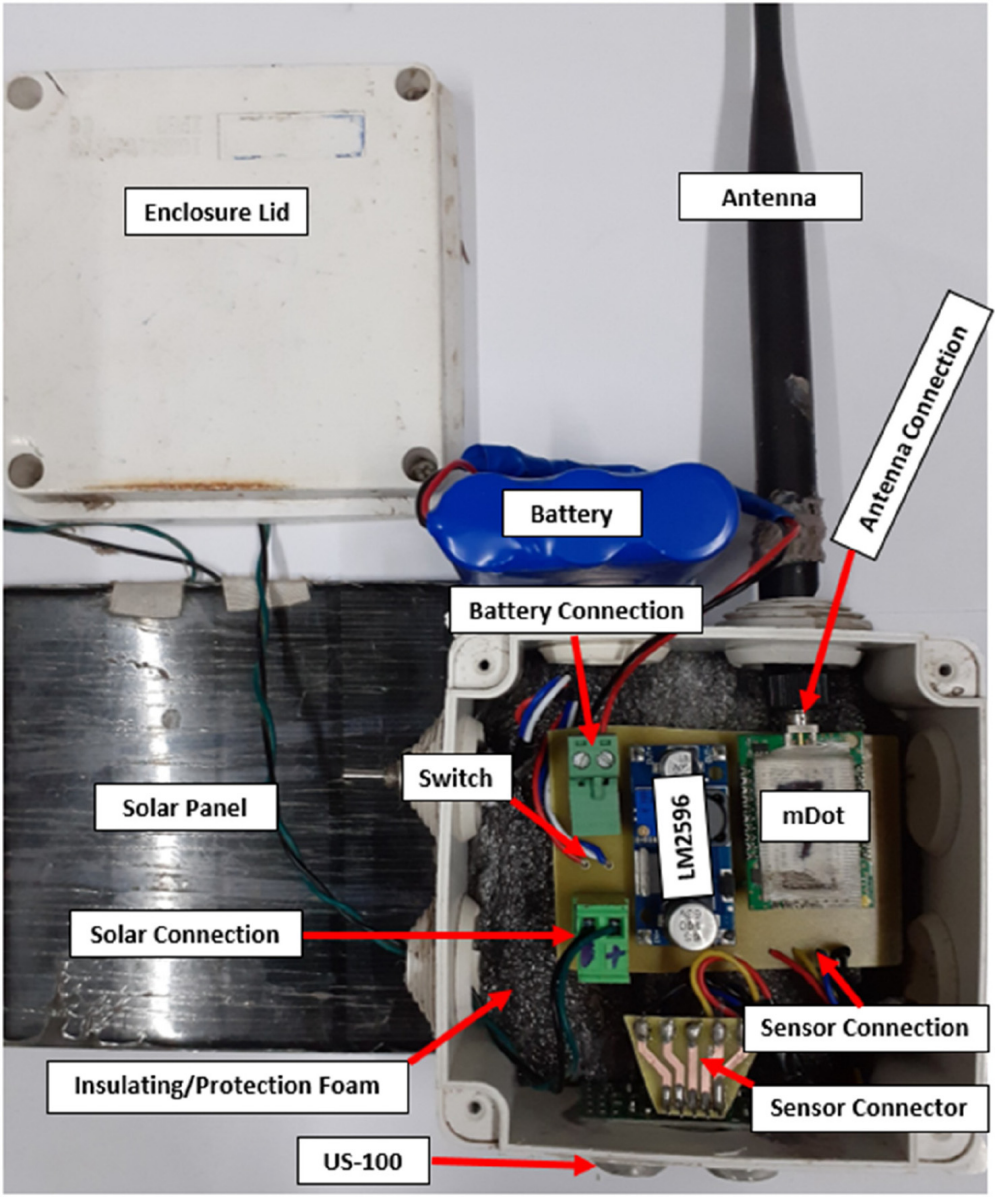
Fully assembled water level monitoring sensor node.

#### Assembled sensor node

Fig. 11 shows a fully assembled sensor node. The battery and the solar cables were connected to female terminal block pieces and inserted to their respective male terminal ports which were already soldered onto the board.

The mDot was mounted on its socket and the antenna was attached to its onboard socket. The solar cable came in through the rubber bungs and so did the antenna. Beneath the circuit board was an insulating foam that isolated the board from the head of the bolt used to fasten the node housing to the deployment support. The sensor and switch extended connections were also finalized. During deployment, the battery was placed on top of the circuit board and then secured with foam to avoid motion. The water proof lid was then firmly secured using the plastic screws at the four corners of the plastic enclosure.

#### Operation instructions

##### The things stack registration

This section outlines the procedures followed in the registration of the sensor nodes on The Things Stack (Version: 3.20.2) before deployment. As a network server, The Things Stack provides an environment where developers can register nodes and gateways. The gateways are used to establish the LoRaWAN networks and the status can be monitored on the Things Stack. The nodes such as the one outlined in this work are used in data collection. The data is transmitted to the server through the network established by gateway. On the network server, developers can also reroute payloads to other permanent databases for analysis, data visualization is also possible.

I. The Thing Stack account login - developers can create an account by following the steps on the official The Things Stack Website.
II. On the account, developers can register gateways. Creation of applications which hold the devices/nodes is also possible.
III. On the application tab, click on “**add application**” to create an application for the water level sensor node. Click on the application to access its page.
IV. Click on “**add end device**” to register the node in the application created. The following steps can be followed:
• Registration - **Manually**
• Frequency plan: **EUROPE 863**–**870 MHz (SF9 for RX2 recommended)**
• LoRaWAN version: **MAC V1.0.4**
• Regional Parameters version: **RP001 Regional Parameters 1.1 Revision A**
I. Click on “**Show advanced activation, LoRaWAN class and cluster setting**” and the following steps can be followed:
• Under **activation** mode: check “**Activation by personalization (ABP)”**
• **Check**: “**Use external LoRaWAN backend servers**” box
• **Generate**: “**DEVICE ADDRESS**”
• **Generate**: “**APPSKEY**”
• **Generate** “**NWKSKEY**” and give the device an “**END –DEVICE ID***”*
• **Under** “**after registration**”, check **“View registered end device”**
• **Finish** the registration by clicking on “**Register end device**”
II. Click on the device registered, click on **General Settings**. Under “**Network layer, LoRaWAN network-layer settings, behaviour and session**” click on the **Advanced MAC Settings panel** and check the “**reset frame counters**” box. Save and exit.
III. Under “Payload formatters” select “**Custom JavaScript formatter**” on the dropdown. Copy the code provided in the design files table (**formatter.js**) and paste it on the payload-formatter code section provided on The Things Network. Save and exit.

## Sensor node operation Instructions. (Firmware upload and operations)

### Installations: Mbed cli to compile code, MS visual studio code or any other code editor, St-link drivers, Tera-term.

I. Run the command prompt and navigate to any other storage folder.
II. Command to import the code/firmware with all the necessary libraries: “**mbed import** **https://github.com/DeKUT-DSAIL/hardwarex-waterlevel2”**. Code also available on the source provided on the design files table
III. Access the files using a code editor. In the “**devices_addresses.h**” file fill out the KEY slots – **Network Session Key, Application Session Key and Device Address Key** – with the KEYs obtained from The Things Stack “**device session terminal**”. In the “**depth.cpp – line 13**” file a developer can choose the time interval between payloads.
IV. Command to compile the code: “**mbed compile -m MTS_MDOT_F411RE -t GCC_ARM**”. The compilation takes about 10 to 20 min.
V. Connect the mDot to the computer using the programming module obtained on purchase. The ST-LINK installation enables the computer to open up the mDot and other mbed enabled devices as mass storage devices.
VI. A bin file is produced and stored in a build folder that is accessible through the file explorer. Flash the code into the mDot by dragging the bin file to it.
VII. Unplug the mDot programmer from the USB port and the mDot thereafter. The programmer notifies a user the flashing is done by turning its programming LED green.
VIII. On the circuit board, before mounting the mDot, confirm the voltage levels in the power supply tracks using a digital meter
IX. After confirmation, plug the mDot on the header pins socket on the circuit board, connect the antenna to its socket and flip the switch. Check TTN terminal whether the device is on and if the payloads are coming in. The mDot does not have a power LED.

#### Deployment

This section gives an elaborate explanation on the deployment process of three sensor nodes that were built using the outlined methods. The sensor nodes were deployed along River Muringato in the lower catchment of the Muringato sub-catchment. Before fitting the sensor nodes with deployment accessories, reconnaissance visits were conducted along the lower catchment section of River Muringato. This deployment site surveys aided in identifying suitable gauging sites along the river. Some of the main influences behind the choice of the gauging locations were, the presence of permanent rigid structures across the channel such as bridges and old tree trunks, type of river flow under the permanent structure (lamina flow preferred), the depth of the river channel at the permanent structure location (sensor limitation (417 cm)) and the type of vegetation surrounding a location (peak solar energy). In the lower catchment area, three suitable locations were marked and prepared for deployment.

**Fig. 12:**
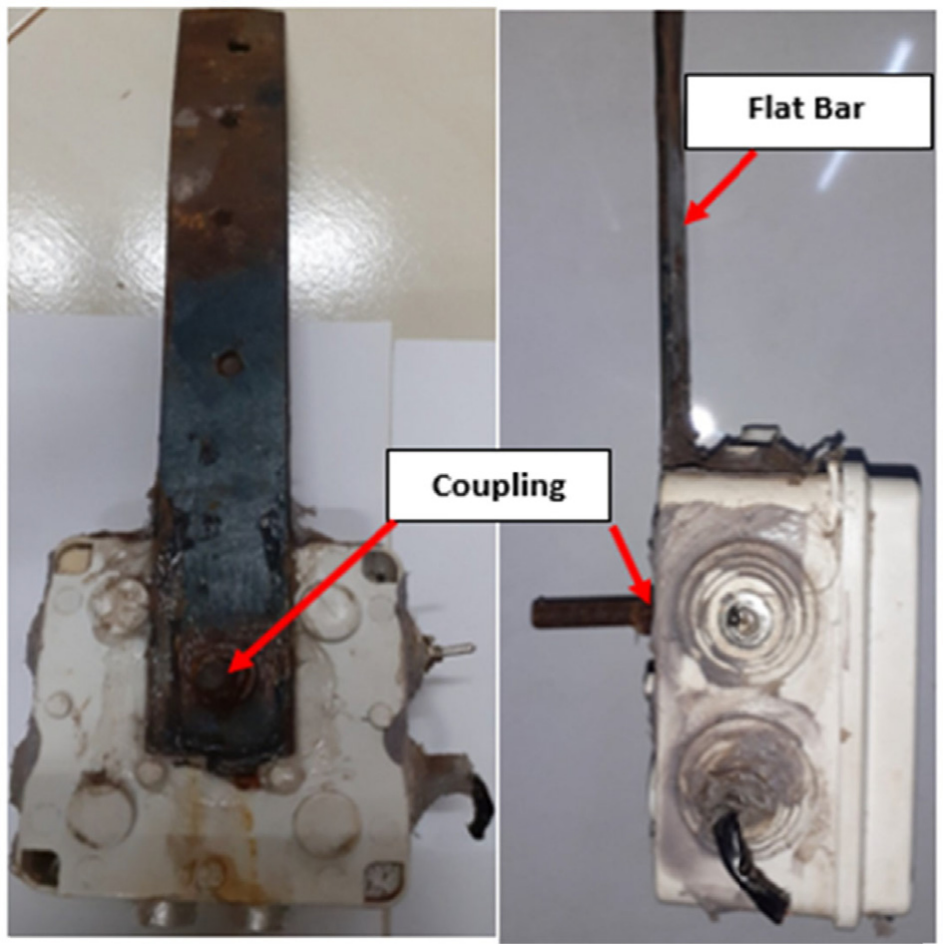
Deployment accessory fitting.

**Fig. 13:**
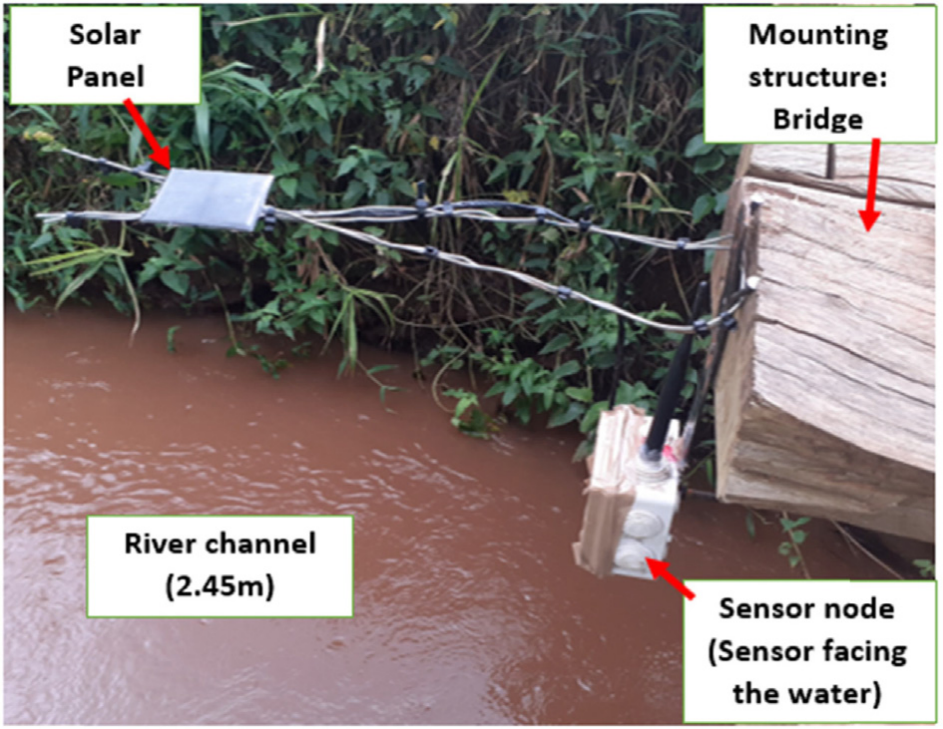
Deployed sensor node (Vertical Deployment Design – 1).

An important point to note in this section is that the external sensing elements had to be at a 90-degree angle with the water surface as shown in Fig. 13. The metallic component attached to the sensor node housing as shown in Fig. 12 had to be designed after a careful assessment of the desired gauging location and an identification of the permanent support structure available since, the shape of the metallic piece depended on these two factors.

One of the deployment designs adopted was a vertical deployment design. The developed sensor node was coupled with a 1ft long, painted mild steel flat bar (1″ by (1/8)″) (M10) as shown in Fig. 12.

The coupling was done using a hex bolt (M9), a nut and a washer. The bolt ran through the back section of the sensor node plastic housing and then through one of the drill holes on the flat bar. Fig. 13 shows the deployed sensor node and the solar placement.

Silicone sealant was applied at the solar cable and antenna entries and also at the flat bar coupling point to prevent water from getting through the unsealed openings. The flat bar was finally nailed to the rigid structure for support (bridge). The deployment design used was chosen because it suited the gauging point.

**Fig. 14:**
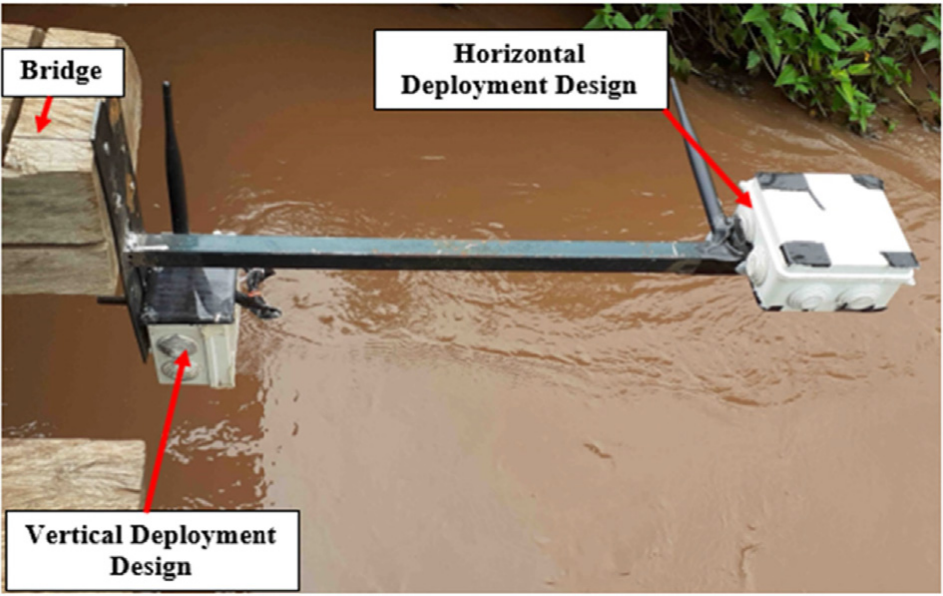
Deployed sensor node (Horizontal Deployment Design – 2).

Fig. 14, shows a horizontal deployment design. This design was also used at one of the two remaining gauging sites. The main advantage of this design was the clearance between the rigid structure and the sensor node. The vertical deployment design also provided some clearance but in case of an unprecedented rise in water level, the sensor would have come into contact with the water surface rendering the sensing element useless. Before deployment, the highest water level mark of the channel was established to determine at what height were the sensor nodes safe and what deployment design was suitable for a particular location.

## Validation and characterization

In this section, is a detailed description of the sensor node power requirements and the importance of the battery voltage data in monitoring the amount of power available for the sensor node during the deployment period. Another highlight in this section is the establishment and analysis of the LoRaWAN network used in data transmission throughout the deployment period. The network description is linked to maps of the deployment location, which aid in capturing the deployment scale and the project scope. Lastly, the water level data collected is presented under section 6.3. All the validation exercises done to prove that the water level data collected was correct (made sense) and the sensor node worked as expected are described in this section.

### Energy consumption modelling

The special Medium Access Control (MAC) protocols used for wireless sensor networks such as LoRaWAN are targeted at energy efficiency, reliability, low access delay and high throughput since sensor nodes have limited power resources [15]. Other objectives of the LoRaWAN technology are self-configuration and reducing the device production cost. The chirp spread spectrum (CSS) modulation used by LoRa enables the adoption of the low power merits which are beneficial in increasing the communication range. Depending on the application, the LoRaWAN protocol spells out three classes available for different power utilization plans and data transmissions layouts. In this study, the device developed was a class A device/node. Class A nodes have the lowest power requirements and can allow bi-directional communication (downlink and uplink). The nodes can also initiate based on their own requirements which are usually stated in the firmware. In a normal operation, each uplink is followed by two downlink messages.

Sleep mode is one of the power saving features utilized in modern microcontrollers. Other known methods are: processor clock speed throttling and power off. To minimize the amount of power consumed by the sensor node, the microprocessor was programmed to acquire and send payloads at 5-minute intervals.

In sleep mode, the controller draws only a small fraction of the current it draws when active. Since the active period is usually between 1 and 3 s long, the average current value is very close to the sleep mode current value and hence the overall amount of power consumed is low. During tests and analysis, the node described was found to draw 2.2 mA during sleep mode and 32.6 mA during payload acquisition and transmission. Table 5 outlines the magnitude of current drawn and projections. Equation (1) highlights the calculation of the average current value which was used in calculating the number of days the device could last without the solar connection.

(1) $$I_{\mathrm{AV}}=\frac{\left[I_{\mathrm{Sleep}}\times t_{\mathrm{Sleep}}\right]+\left[I_{\mathrm{Active}}\times t_{\mathrm{Active}}\right]}{\left[t_{\mathrm{Active}}+t_{\mathrm{Sleep}}\right]}=\frac{\left[2.2mA\times 300s\right]+\left[32.6mA\times 3s\right]}{\left[3s+300s\right]}=2.5009mA$$

**Table 5:** Sensor Node Power Figures.

Sleep mode current I_sleep_	Peak Active mode current I_active_	Average current Iav	Sleep mode time t_sleep_	Active mode time t_active_	Number of days without solar
2.2 mA	32.6 mA	2.5009 mA	300 s	3 s	110 days

To get the number of days the system would last without being charged using the solar panel, the battery capacity figure (6600 mAH) was divided by the average current value (2.5009 mA) = 110 days. The power values shown on Table 5 show that the sensor node described was power efficient and could last for years in the field if a reliable solar power source was connected. Three sensor nodes were deployed along River Muringato. The river flows through the Muringato watershed in Nyeri county, Kenya and is the main drainage system in the watershed. Data was acquired from the three nodes for more than 18 months.

#### Battery voltage data

Over the 18-month period, the stage monitoring node sent payload packets which constituted the battery voltage data and the water level data. The battery voltage data was obtained using the layout described under “Battery voltage sensor” Section. Using the battery voltage data, it was possible to analyse the effectiveness of the limited amount of power coming in from the solar panel. In a normal operation, if the battery voltage level fell below the cut-off voltage (2.8 V), the system powered down due to the inadequate power and voltage levels. If the solar power was enough to charge the battery, the node was powered back on to resume data collection. Over the 18-month period the battery levels of the three deployed nodes did not drop below 3.4 V due to the solar top-up system. Fig. 15a, Fig. 15b show battery voltage – against - time plots of two sensor nodes from 21st January 2022 to 17th February 2022. Fig. 15a shows the solar panel was charging the battery regularly since the voltage did not drop over time. Fig. 15b reveals that the solar panel was not charging the battery since the voltage declined over time; this scenario was simulated by a deliberate solar disconnection for the stated time period. In normal practice, factors such as solar blockage, solar panel malfunction and open solar circuit may lead to such behaviour. More detailed studies based on these plots can be done to improve deployments on a large scale.

**Fig. 15a:**
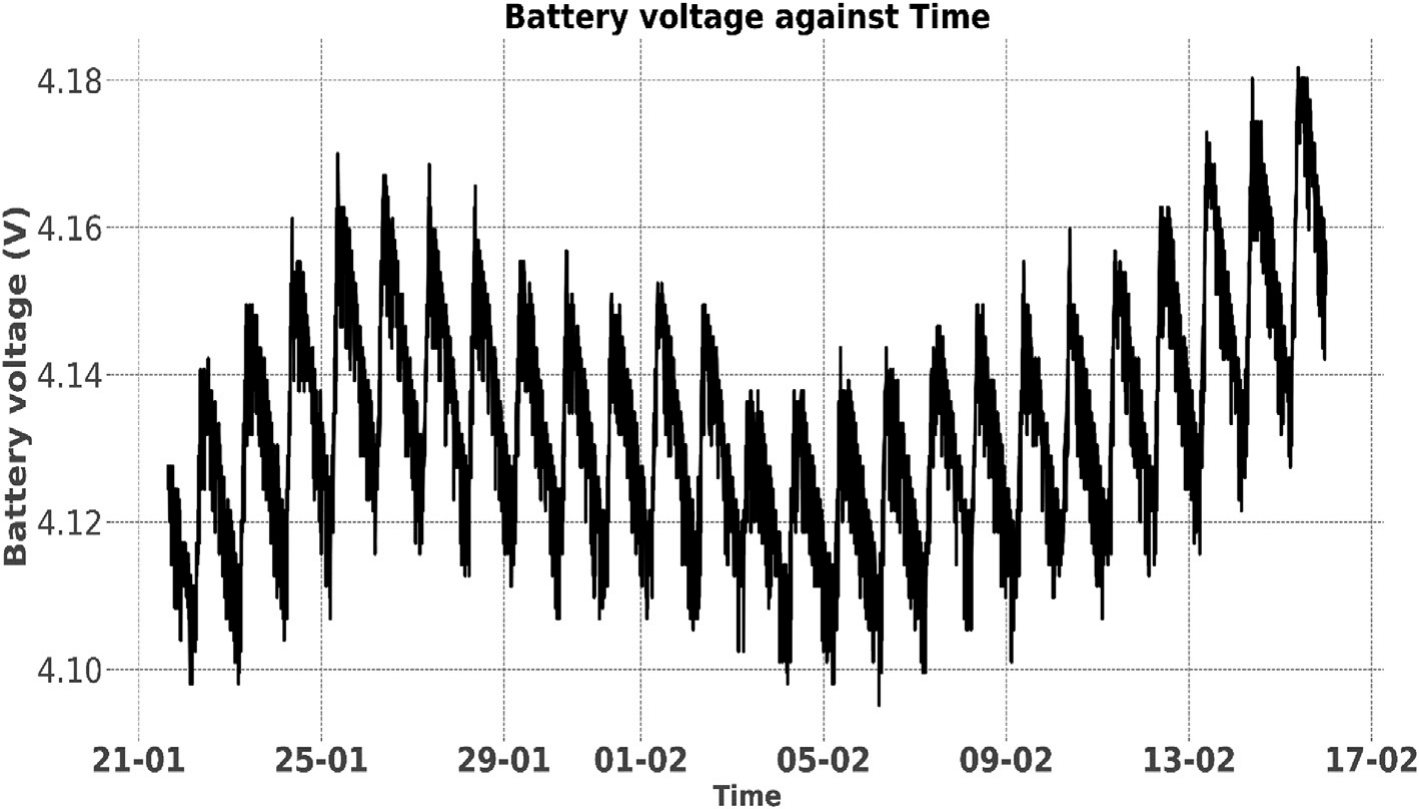
Battery voltage Vs. time (Charging System Connected).

**Fig. 15b:**
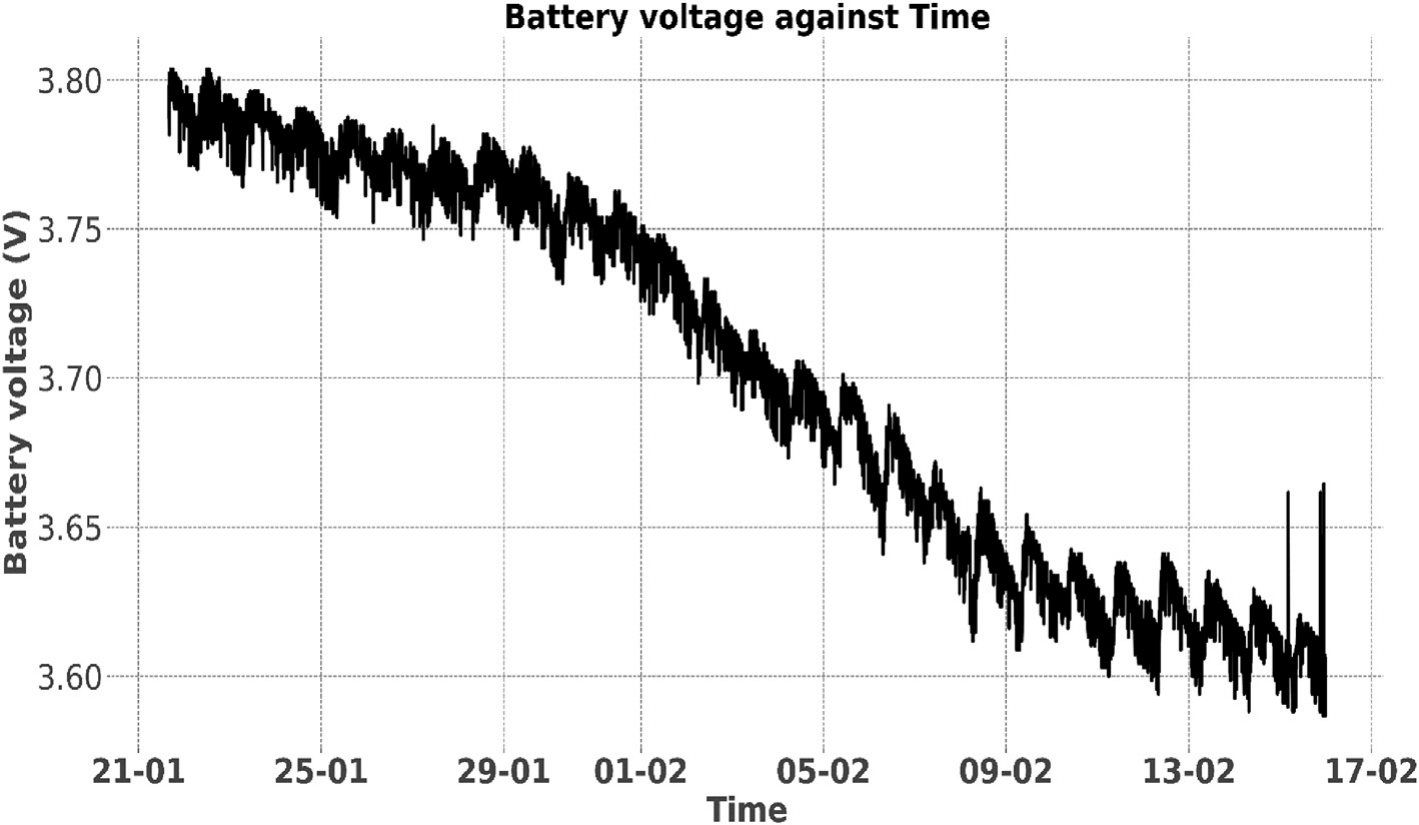
Battery Voltage Vs. Time (Charging System Disconnected).

### LoRaWAN network establishment

This section is a detailed outline of how the LoRaWAN network utilized by the developed sensor nodes was set up and analysed. The network setup and sensor node deployment were application scenarios in a specific hydrological region of the Muringato watershed. The deployed sensor node discussed in Section 5 was utilizing a LoRa network that was established to cover a section of the target river (River Muringato – Nyeri county, Kenya), to transmit data to the network server. The network was established by deploying two gateways/concentrators at different heights. Table 6 shows the gateway specifics.

**Table 6:** Gateway Specifics.

Gateway	Gateway 1	Gateway 2
Type	Kerlink Wirnet Station	Multitech Conduit
Deployment Height	21 m above ground	46 m above ground
Gateway antenna type	3dBi	3dBi
Signal strength from the deployed nodes (1 km away)	Mean RSSI = -113dBm Mean SNR = 7.2	RSSI = -95dBm SNR = 10.32

To map out the length of the river section covered by the network established, a radio mapping (network coverage test) exercise was conducted. The task involved the evaluation of RSSI and SNR values of test sensor nodes at different points in the Muringato watershed and marking out the test locations on a map. The test points considered were at different distances from the getaways in different directions.

RSSI refers to the signal power that is received by the gateway/receiver in milli-watts (mW), and it is measured in dBm. How clear a receiver can “hear” from a sender/node can be measured using this value. Received signal strength indication, i.e., RSSI, is usually a negative value; hence, the signal is better when it is closer to 0. The value ranges of typical LoRa RSSI are −130 dBm to −30dBm.

The difference in RSSI as shown in Table 6 was brought about by the difference in height. The Multitech conduit, at 50 m, had a Line of Sight – less obstructions – advantage over the Kerlink gateway, which was just 21 m high. The line-of-sight advantage and less obstructions made the Multitech mDot have a lower link budget. A link budget is a consideration of all the power gains and losses a signal goes through in a wireless communication system or any other telecommunication system.

The averaged packet loss for the deployed nodes was between 1% and 5%. To get an insight into the percentage packet loss value, the tally of all data points obtained from one sensor node from 25**th** to 29**th** march 2022 was considered. Out of the 1440 data points expected, 1401 data points were obtained, which in turn represented a 2.708% percentage loss. Fig. 16a, Fig. 16b show the expected variations of the received signal strength values for the Multitech Conduit gateway and the Kerlink Wirnet station gateway at different test distances respectively.

**Fig. 16a:**
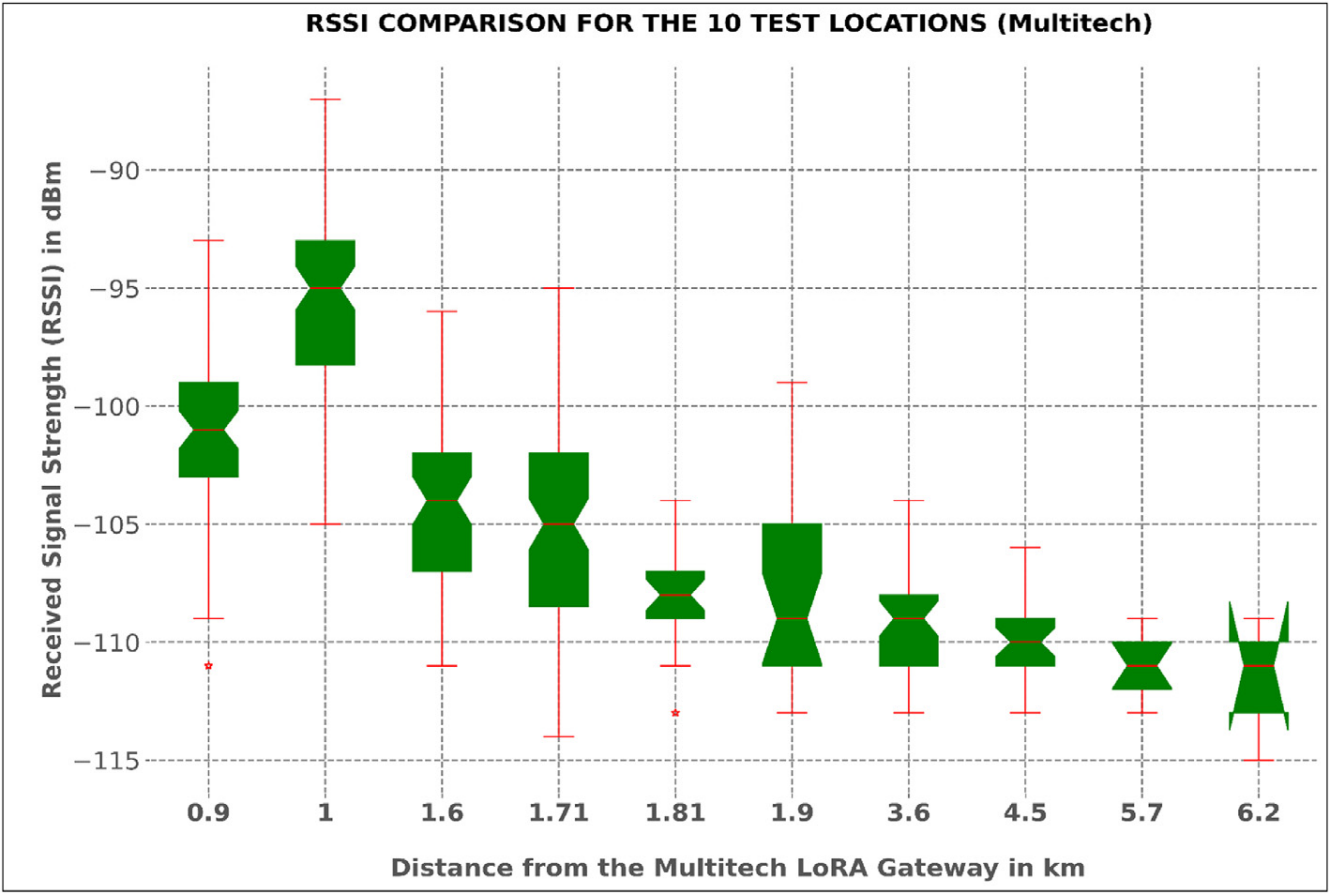
The Received Strength Plots for the 10 Test Locations (Multitech Gateway).

**Fig. 16b:**
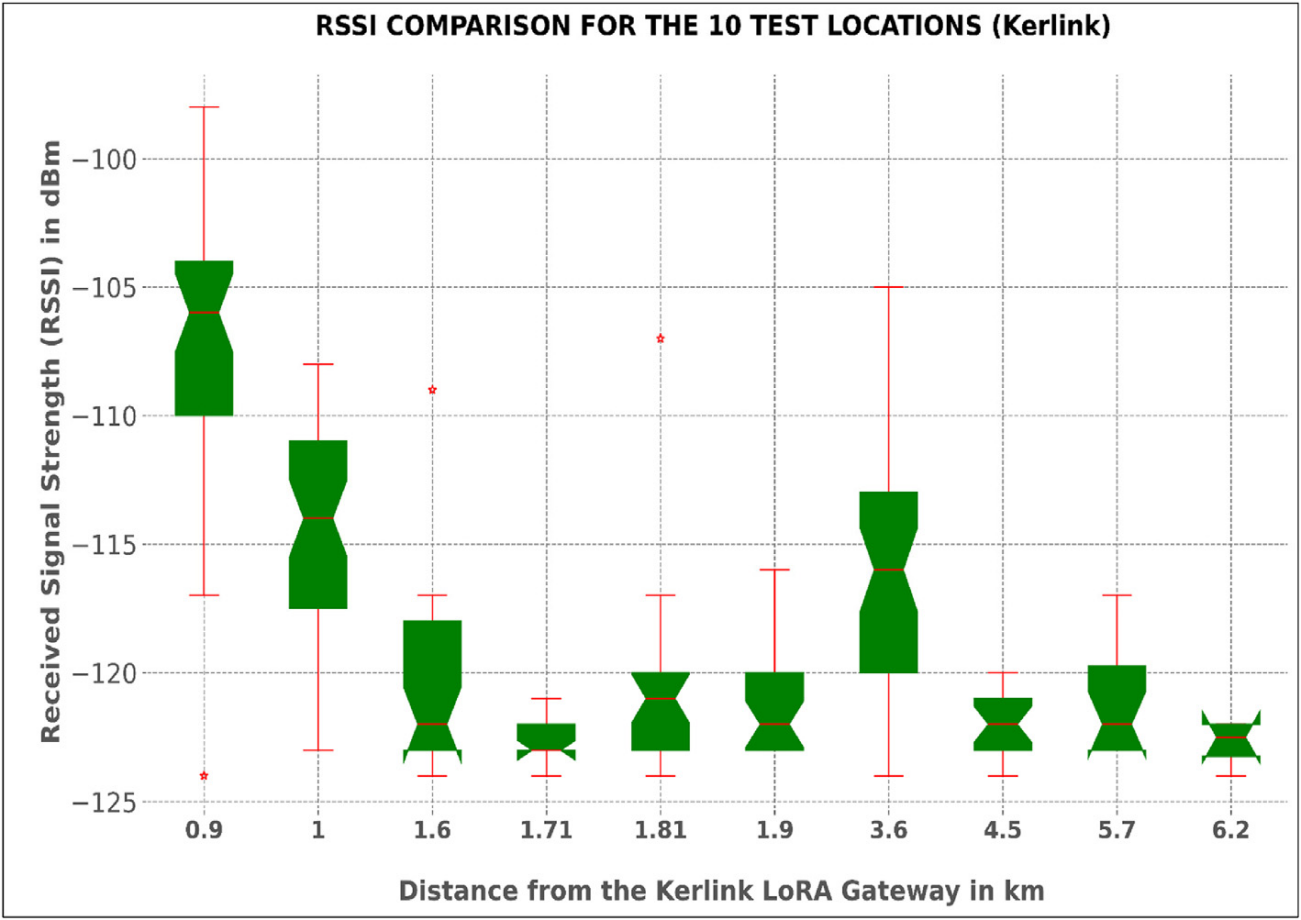
The Received Strength Plots for the 10 Test Locations (Kerlink Gateway).

The box plots provide a graphical examination of the RSSI for each of ten test locations for the gateways. During the coverage test, the test sensor node was held at a height of 1 m above ground and powered to send dummy payloads with the RSSI data at 15 sec intervals for 10 min, at each test location. This translated to 40 RSSI sample data points for each location represented by the box plots. The coverage test was done for both gateways. Based on the plots, there was a general non-linear variation of the median positions of the RSSI, usually determined by various parameters, which include free space loss, shadowing, reflection and transmission, diffraction, among others. The developed nodes were deployed 1 km away from the gateway (Figure 16-a and b). The signal strength at this location was practically adequate and this also led to a low packet loss.

**Fig. 17:**
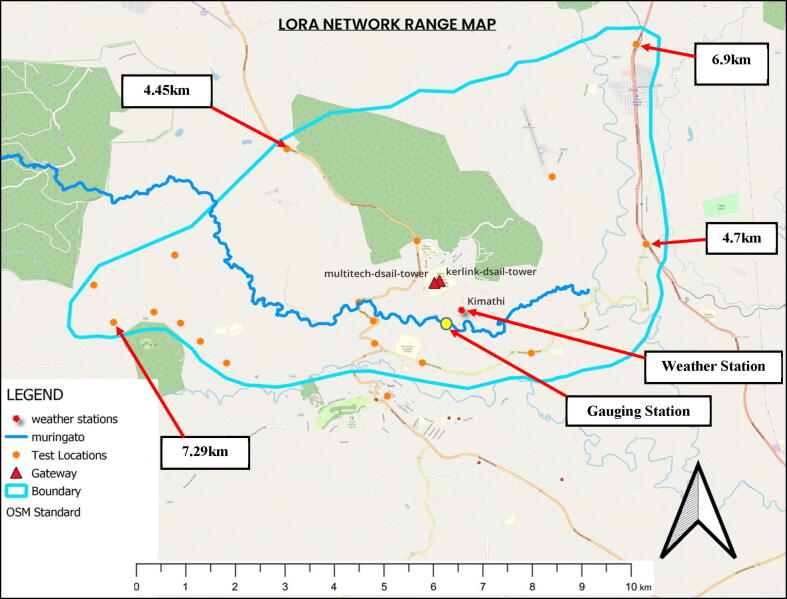
Radio Mapping Results (Gateways – Red Triangle) - (Boundary in Cyan) - (Deployment- Location in Yellow). (For interpretation of the references to colour in this figure legend, the reader is referred to the web version of this article.)

Shown in Fig. 17, is a map that represents the area covered by the established network in the Muringato catchment (yellow: deployment location, orange: Network test locations, Red: Gateway location). The deployed gateways were practically at the centre of the area covered by the established network. The gateways were strategically installed at different heights for maximum coverage. The radio mapping/coverage test began at areas closer to the gateways followed by areas further away as indicated by the test location marks on the map. The closest test location was 0.9 km away whereas the furthest was 7.29 km from the concentrators. As expected, the network strength at a particular point was inversely proportional to the distance from the gateway hence, the coverage at 7.29 km was weak compared to the coverage 1 km away.

Compared to the western section of the area covered by the established network, the eastern section was wider, this was brought about by the difference in terrain. As expected and desired, a significant section of the Muringato River was inside the network boundaries as indicated. The yellow mark on Fig. 17 indicates one of the main deployment areas where two of the water level monitoring devices were deployed. The area was approximately 1 km away from the gateways and was in the Line of Sight (LOS) with one of the gateways (46 m high – Multitech Conduit) which in turn translated to strong network coverage of the area. Also shown in Fig. 17, is a weather station (TAHMO station Kimathi). The time series rainfall data sets from this weather station and others in and around the Muringato sub-catchment were used in validating the water level data collected. The weather station was about 400 m away from the gauging location as shown.

**Fig. 18:**
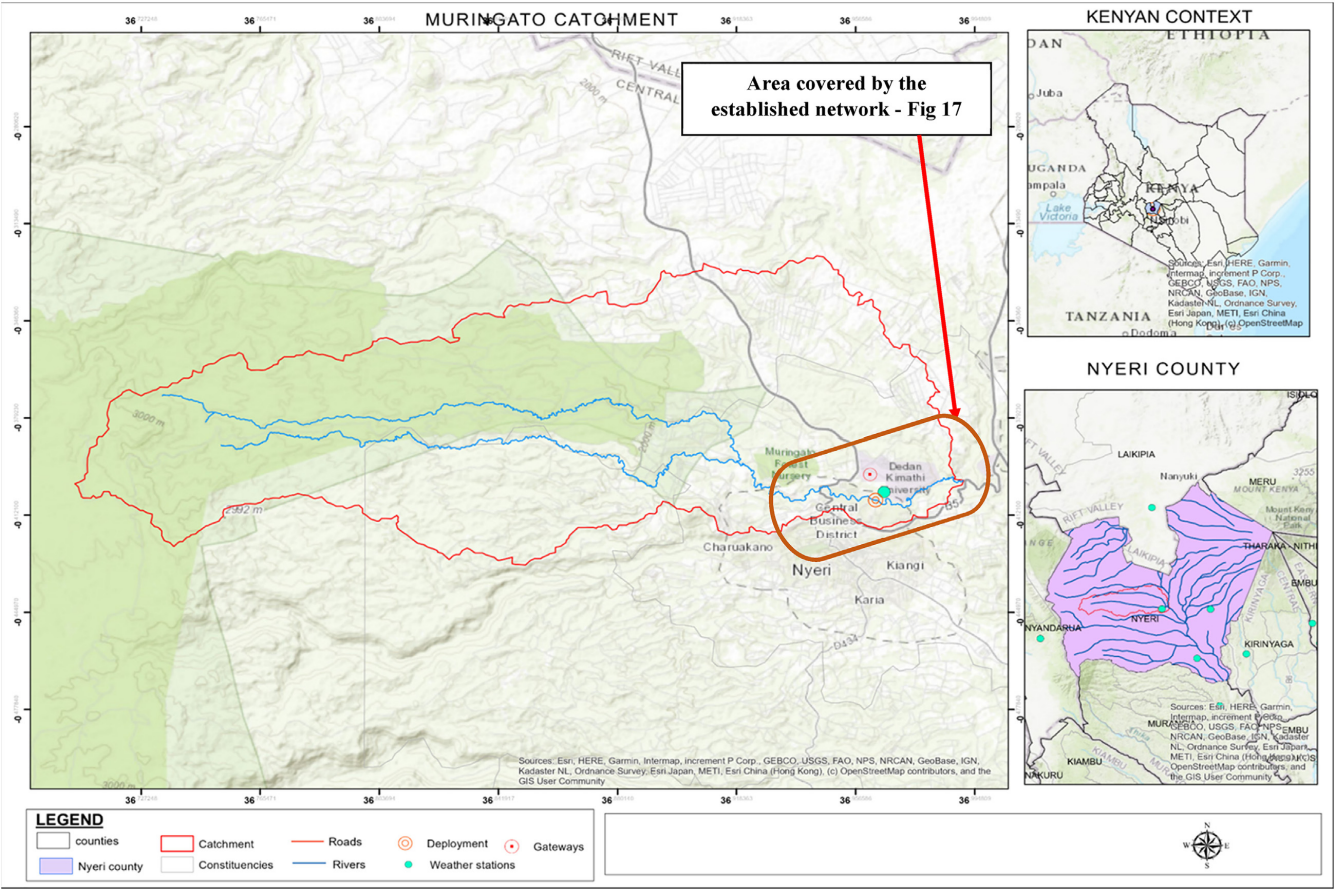
Muringato Catchment Under Study.

Fig. 18 is a map representing the entire Muringato sub-catchment/watershed under study. The main components on the map are; the river Muringato in blue, the area covered by the established LoRaWAN network with respect to the entire catchment and also the sub-catchment size and boundaries in the Nyeri county context and the Kenyan context. The sub-catchment covers a total area of 225 square kilometres and possess the common characteristics of catchment areas. The steep highlands to the west are covered by the Aberdare Forest. The forest is a highly protected resource and many of the tributaries that join to form the river under study originate here. The lower lying areas to the south-east are under agriculture and settlement hence the river is a vital resource in these areas. The source of the Muringato (Aberdare Forest) is at an approximate elevation of 3000 m above sea level (ASL). The ASL of the lower catchment as the river exits the water shed and drains into another major river is 1700 m hence the average river gradient across the sub-catchment is 1.23%.

### Water level data

This section presents the water level data collected by one of three sensor nodes that were deployed, and the validation methods used in proving its relevance. Another highlight in this section is the sensor range test which was used in determining the object detection range of the sensor before deployment. In the first validation method/procedure, the water level data collected is compared to manual water level measurements taken using a graduated gauging rod and the results are highlighted in Section 6.3.2. In the second validation procedure, the water level data is matched with rainfall time series sets from weather stations in and around the catchment area and the results are presented in Section 6.3.3.

#### Sensor range test

**Fig. 19:**
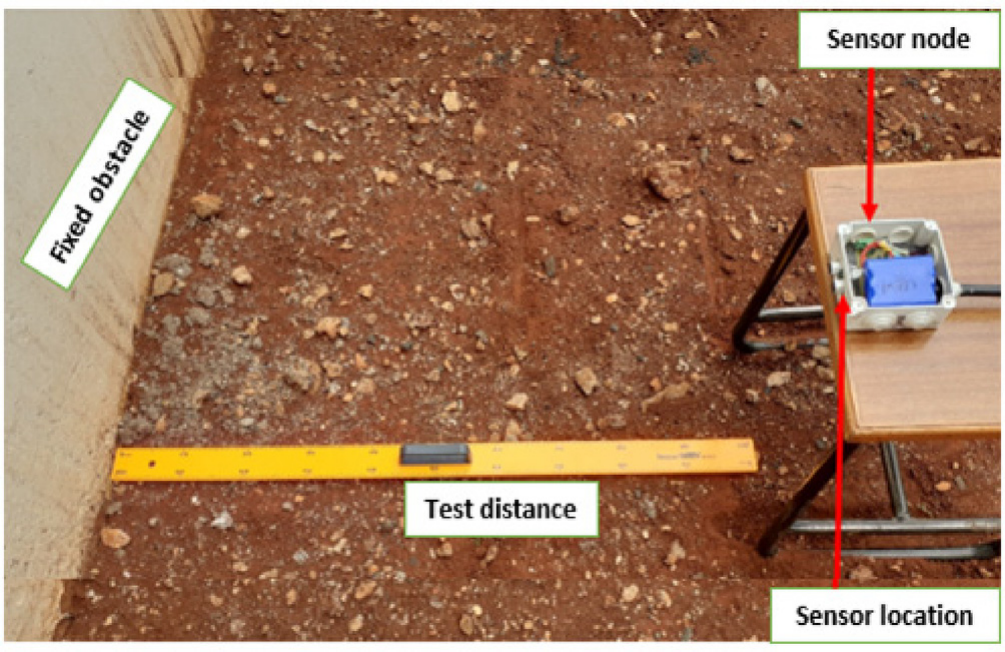
Sensor Range Test Setup.

Before deployment, the 3 sensor nodes developed were subjected to a range-test experiment. The outdoor test was done to establish the maximum object detection (measurement) range for each node. The test was done outdoors to simulate the deployment conditions and to provide exposure to weather elements. Testing was done at different test distances (100 cm, 200 cm, 300 cm, 417 cm) as shown in Fig. 19. For every test distance the nodes were programmed to send data after 30 s and the test time at every test distance was 35 min. This setup generated 70 data samples for every test range. The maximum test range was 417 cm since the nodes were collecting erroneous measurements for ranges above this value. After the test, the data collected at each test distance was analysed. Table 7 shows averages of the data collected at each test distance compared to the real measurements. Based on these results, the sensor nodes measurement error was negligible. Since River Muringato channel depth at gauging station 1 was 2.08 m, at gauging station 2 was 2.25 m and at gauging station 3 was 2.45 m and also since the US-100 operated well between 10 cm and 350 cm, it was concluded that the sensor nodes were fit for deployment at the identified locations.

**Table 7:** Sensor Limit Test Result.

Test distance in cm	Mean sensor output in cm
100	100.30
200	200.13
300	300.04
417	417.76

#### Validation 1- Manual Measurements

**Fig. 20:**
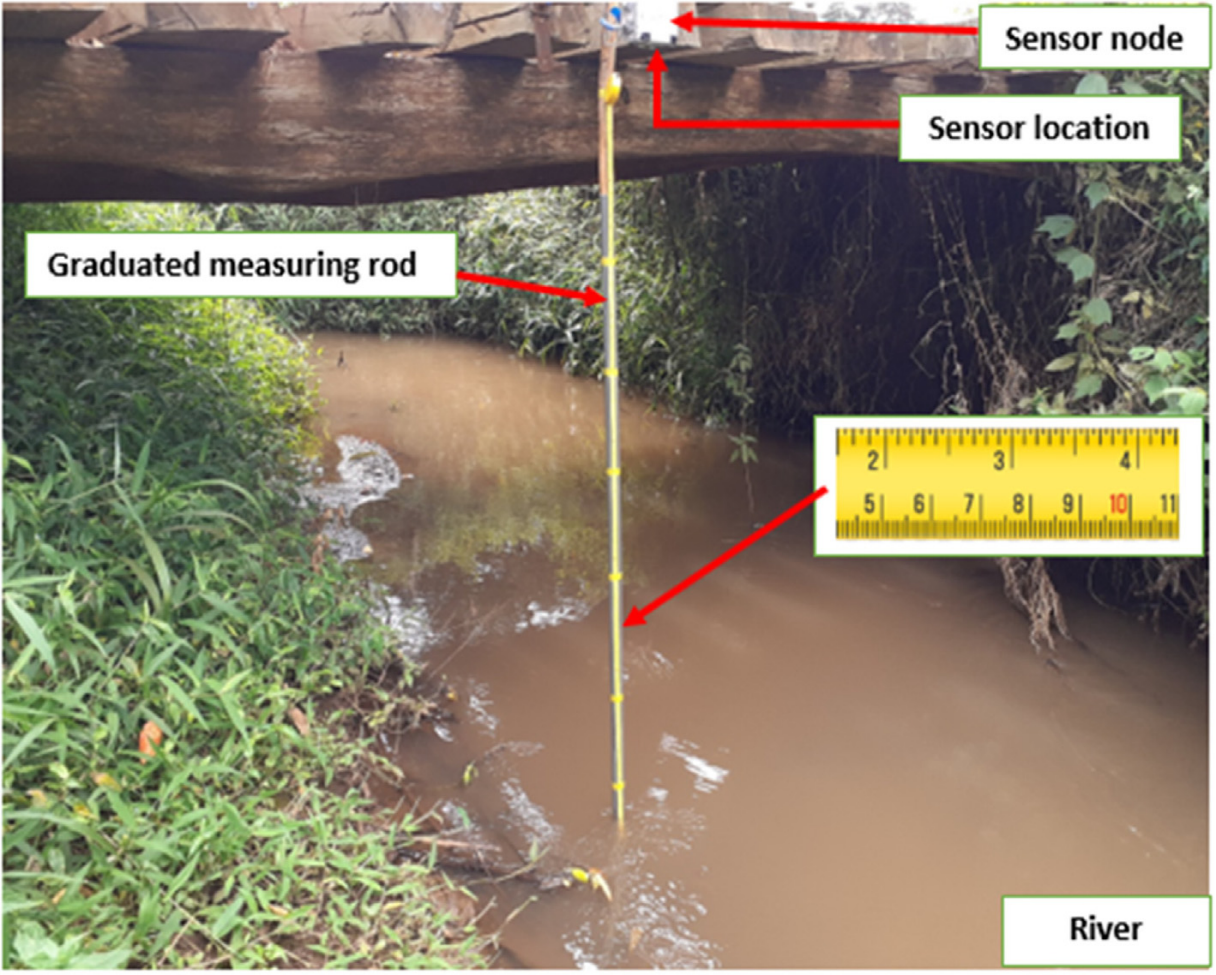
Manual Measurement Setup.

To validate the data acquired, manual measurements were taken at regular intervals and recorded on a spreadsheet. These measurements were made using a calibrated staff gauge. The recorded data was compared to the sensor data which had a time stamp corresponding to the manual measurement timestamp. Fig. 20 shows a manual measuring setup at one of the gauging locations. The manual measuring was done over a two-month period.

**Fig. 21:**
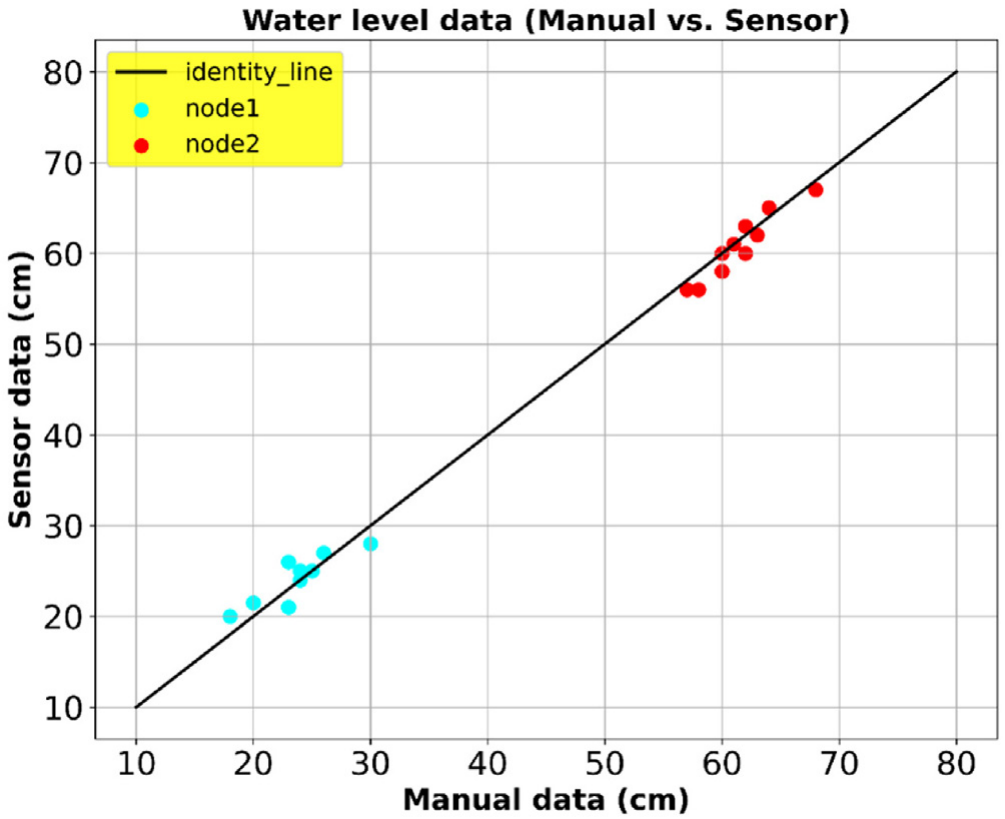
Manual Data Vs. Sensor Data.

Fig. 21 shows the correspondence between the manual water level readings and the sensor readings. The cyan cluster shows validation comparison for one of the sensor nodes deployed at a wide cross section area along river Muringato. The red cluster shows a comparison for another sensor node that was deployed at a deeper gauging location. From Fig. 21 the computed RMSE representing the error margin between the measured values and the sensor values for node 1 and node 2 were 1.71 and 1.30, respectively. The analysis done provided prove that the deployed sensor nodes were working as expected.

#### Validation 2: Rainfall data

The water level data obtained from the sensor node was also compared to rainfall data acquired from rain gauges in the Muringato watershed. Correlation analysis was conducted between the 2021 water level data time series set and seven rainfall time series sets from different parts of the watershed. Five out of the seven time series sets were in positive correlation with the water level set indicating that the sensor nodes developed were working well and the data obtained was relevant. The two rainfall time series sets that did not match the water level data indicated that rainfall in some parts of the watershed was not contributing towards the change in water level (stage response) in the river.

The rainfall data was obtained from Trans African Hydro-meteorological Observatory (TAHMO). Shown in Fig. 22a is a comparison plot between water level daily mean values (line graph) and rainfall daily measurements in mm from TAHMO station Kibugu (bar graph), which is located around the Muringato watershed. The plot shows a high degree of correlation between the two datasets which is highly probable when carrying a comparison study between rainfall and stage. It also indicates that the sensor nodes were giving correct data that is relevant for the catchment under study.

**Fig. 22a:**
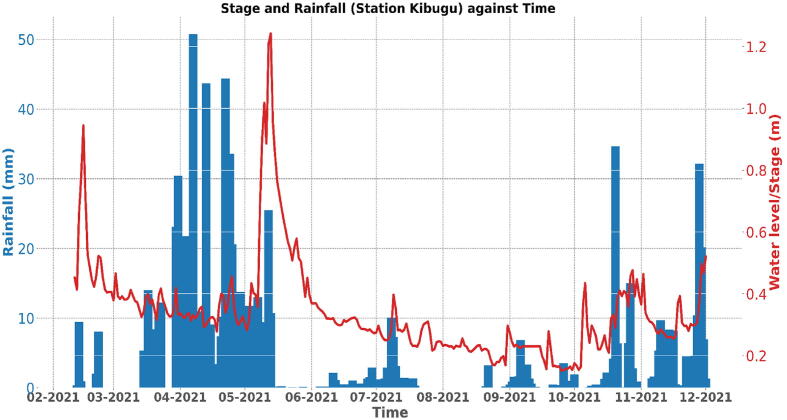
River Muringato Water-Level Vs. Rainfall (Kibugu Station − 2021).

Fig. 22b also shows a comparison plot between water level daily mean figures (line graph) and rainfall daily measurements in mm from TAHMO station Kimathi (bar graph) – shown in Fig. 17 -which is located in the river Muringato lower catchment area. This plot also shows a high degree of correlation between the two datasets which indicated the data obtained from the nodes was reliable and useful.

**Fig. 22b:**
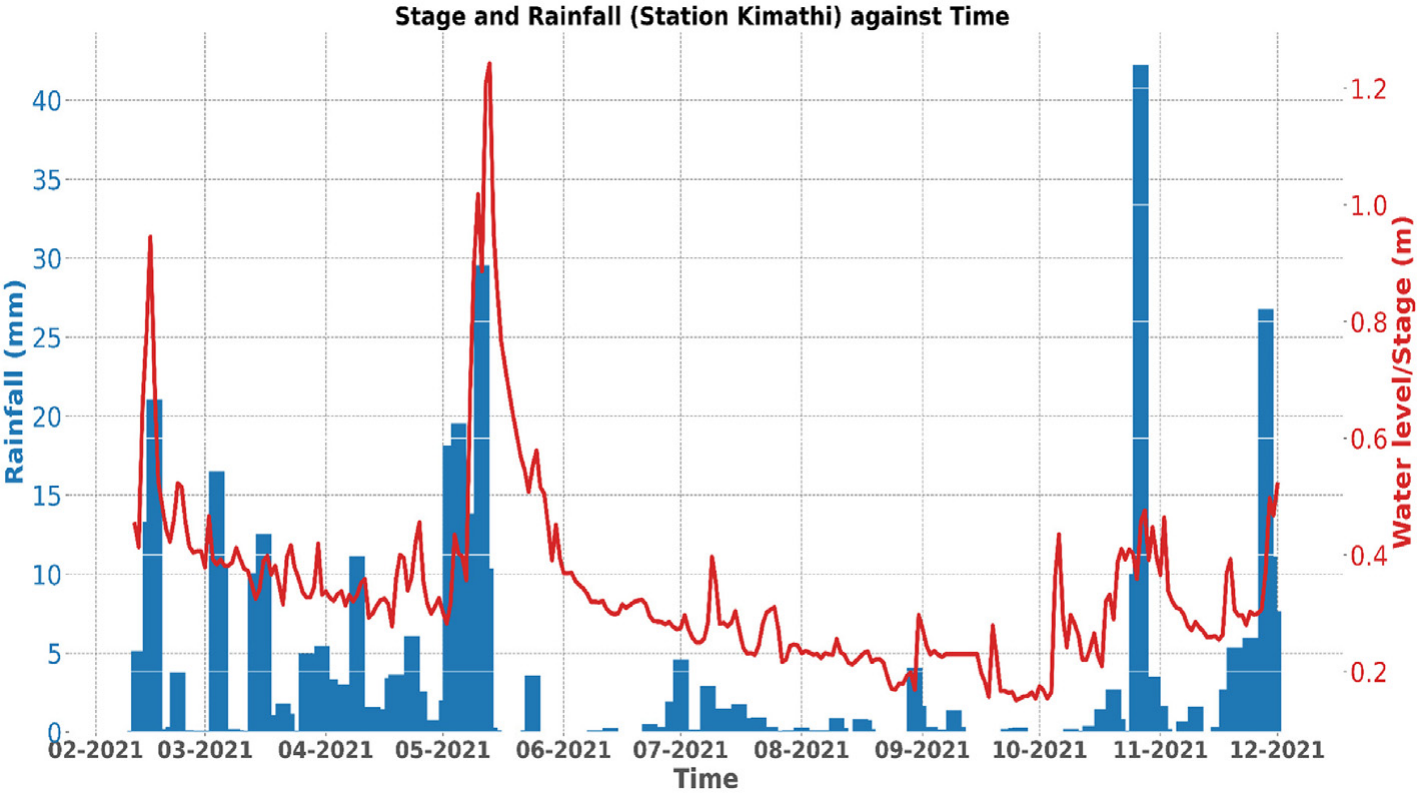
River Muringato Water-Level Vs. Rainfall (Kimathi Station − 2021).

Over the years, engineers and hydrologists have been developing models to establish the relationship between catchment variables such as stage, discharge and precipitation. From a generic point of view, a researcher might assume that the relationship between rainfall and stage in a catchment is direct; an increase in rainfall translates to an increase in stage; but this relationship is affected by other catchment variables such as vegetation cover, soil type, soil moisture content and temperature. Hence, an increase in rainfall might not result in an increase in stage.

The amount of rainfall that gets into a river is often referred to as effective rainfall. If the catchment takes up the larger share of the rainfall that falls on it, the amount of effective rainfall getting into the stream is little, hence the stage peaks will not be as pronounced as the rainfall peaks. The relationship between rainfall and stage is also affected by the distribution of rainfall in the catchment. The rainfall plots/patterns created from rainfall data collected from different weather stations might not correspond to the stage plots/patterns created from stage data collected at different locations along a stream. Human activities such as agriculture along rivers also contribute to the change in stage patterns in rivers. Large farms along rivers, through irrigation systems can at times draw large amounts of water from these rivers resulting in diminishing stage levels.

## Conclusion

To study the impact of increased demand for water, reduced rainfall due to climate change and catchment degradation, large scale monitoring of river water level patterns is necessary. The result of the monitoring is water level data that can be processed and analysed using modern tools such as machine learning and data science. The processing and analysis can be aimed at catchment area analysis (diagnosis) and the discovery of conservation flaws. Thereafter, conservation-strategy improvements can be suggested. Also, with the inclusion of other relevant catchment area datasets such as rainfall and satellite imagery of the vegetation cover in such studies, the relationships between catchment variables can be investigated and future catchment scenarios such as floods and the drying up of a river channel can be predicted. Traditional methods of monitoring water level along rivers which have been developed over the years are expensive, bulky and may come with burdens that relate to river obstruction and pollution. Thus, cheap, flexible and efficient monitoring methods that improve on the limitations of the traditional methods are required by engineers, hydrologists and researchers at large. The system established in this paper is capable of monitoring water level along rivers remotely via wireless sensor networks. The sensor node is small and has been designed using locally available components which make it accessible to researchers. The sensor node accessory such as the solar panel, is able to keep the node running for a long time without maintenance. The battery voltage sensor is also crucial in studying the power consumption of the device and making proper device-power adjustments. It is hoped that the devices outlined will open up other areas of research by enabling widespread deployment of stage monitoring devices.

## Declaration of Competing Interest

The authors declare that they have no known competing financial interests or personal relationships that could have appeared to influence the work reported in this paper.
